# 5-deoxy-rutaecarpine protects against LPS-induced acute lung injury via inhibiting NLRP3 inflammasome-related inflammation

**DOI:** 10.3389/fphar.2025.1522146

**Published:** 2025-01-28

**Authors:** Jinque Luo, Xin Li, Li Zhang, Meijing Deng, Jieyang Zhao, Jinghuan Zhang, Wenyu Tang, Qinghua Guo, Ling Wang

**Affiliations:** ^1^ Hunan Provincial Key Laboratory of the Research and Development of Novel Pharmaceutical Preparations, “The 14th Five-Year Plan” Application Characteristic Discipline of Hunan Province (Pharmaceutical Science), College of Pharmacy, Changsha Medical University, Changsha, Hunan, China; ^2^ Hunan Provincial University Key Laboratory of the Fundamental and Clinical Research on Functional Nucleic Acid, College of Pharmacy, Changsha Medical University, Changsha, Hunan, China; ^3^ The National and Local Joint Engineering Laboratory of Animal Peptide Drug Development, College of Life Science, Hunan Normal University, Changsha, Hunan, China; ^4^ Department of Emergency, Hunan Provincial People’s Hospital, The First Affiliated Hospital of Hunan Normal University, Changsha, Hunan, China

**Keywords:** 5-DR, acute lung injury, lipopolysaccharide, inflammation, NLRP3 inflammasome

## Abstract

**Introduction:**

Acute lung injury (ALI) induced by lipopolysaccharide (LPS) is a significant medical condition characterized by severe pulmonary inflammation and tissue damage. NLRP3 inflammasome-driven inflammation is essential in ALI pathogenesis, inspiring novel therapeutic strategies that focus on NLRP3 and inflammation. In this study, we investigated the therapeutic potential of 5-deoxy-rutaecarpine (5-DR), a rutaecarpine derivative, in attenuating LPS-induced ALI.

**Methods:**

In this study, we evaluated the effects of 5-DR treatment in mice exposed to LPS, lung tissues, bronchoalveolar lavage fluid, and serum were collected for analysis. LPS-stimulated J774A.1 mouse macrophages were used to further investigate the anti-inflammatory effects of 5-DR *in vitro*. Various techniques including histopathology, Western blotting, and luciferase reporter assay were employed.

**Results:**

5-DR treatment significantly reduced lung edema, inflammatory cell infiltration in mice with LPS burden, and reduced the levels of inflammatory mediators like interleukin-1β in the mice and in LPS-stimulated J774A.1 mouse macrophages. Further western blotting analysis showed 5-DR decreased the levels of NLRP3, cleaved caspase-1, and mature IL-1β in mice and J774A.1 cells exposed to LPS. Additionally, NF-κB pathway activation significantly diminished the inhibition of the NLRP3 inflammasome by 5-DR.

**Discussion:**

Our findings highlight the therapeutic potential of 5-DR as a promising candidate for treating LPS-induced ALI, offering insights into its underlying mechanism that targets NLRP3 inflammasome-mediated inflammation.

## Graphical Abstract

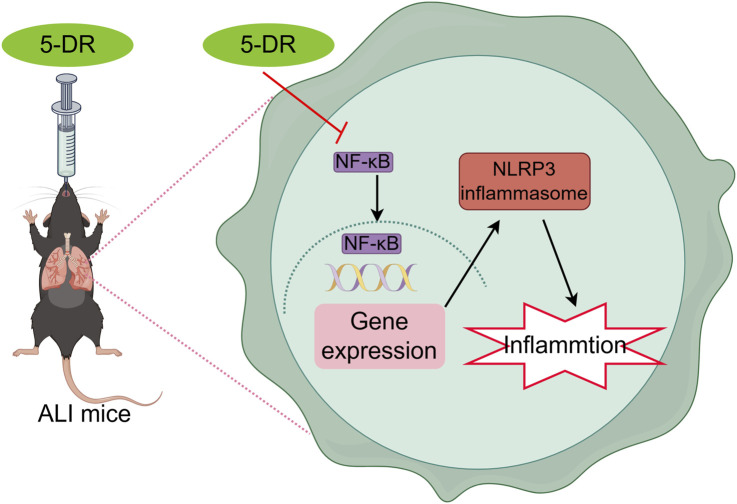

## Introduction

Acute lung injury (ALI) and its more severe form, acute respiratory distress syndrome (ARDS), are life-threatening conditions characterized by acute respiratory failure and widespread inflammation within the lungs (Li G. X. et al., 2022). ALI can be triggered by various insults, including sepsis, trauma, and pneumonia, with bacterial endotoxin lipopolysaccharide (LPS) being a common inducer of lung inflammation (Tsikis et al., 2022). Despite advances in critical care medicine, the mortality rate associated with ALI remains high, underscoring the urgent need for effective therapeutic interventions (Latronico et al., 2024; Cui et al., 2022). Currently, its clinical treatment mainly includes protective mechanical ventilation, with no specific drug for its treatment (Ajibowo et al., 2022), increasing attention has been focused on developing novel pharmacological agents with anti-inflammatory and cytoprotective properties for treating ALI.

In the intricate landscape of ALI, macrophages stand as pivotal orchestrators of the immune response, playing a dual role in both the initiation and resolution of inflammation (Ye et al., 2022). Central to this inflammatory process is the NOD-like receptor family, pyrin domain-containing 3 (NLRP3) inflammasome, a multiprotein complex intricately involved in sensing danger signals and orchestrating the inflammatory cascade (Wei et al., 2020; Yi et al., 2023). Upon sensing danger signals, such as pathogen-associated molecular patterns (PAMPs) or damage-associated molecular patterns (DAMPs), NLRP3 assembles into a multiprotein complex, culminating in the activation of caspase-1 and the subsequent cleavage and release of pro-inflammatory cytokines IL-1β and IL-18 (Chen et al., 2023; Li C. et al., 2022). This cascade of events amplifies the inflammatory response, exacerbating lung injury and compromising respiratory function (Liu et al., 2022). Understanding the intricate crosstalk between macrophages, NLRP3, and ALI holds profound implications for therapeutic interventions. Targeting macrophage activation or NLRP3 inflammasome signaling pathways represents promising avenues for mitigating inflammation and tissue injury in ALI. By modulating NLRP3 inflammasome activity or blocking its downstream effectors, novel treatments may be devised to attenuate inflammation, preserve lung function, and improve outcomes in patients with ALI.

Rutaecarpine (RUT), a bioactive alkaloid derived from the Chinese herb *Evodia rutaecarpa*, has garnered attention for its diverse pharmacological activities, including anti-inflammatory, antioxidant, and vasodilatory effects (Ko et al., 2007; Heo et al., 2009; Tian et al., 2019; Su et al., 2022). Studies have demonstrated the ability of RUT to modulate various inflammatory pathways and cellular processes implicated in the pathogenesis of ALI, including the inhibition of pro-inflammatory cytokine production, suppression of oxidative stress, and enhancement of endothelial barrier function (Li et al., 2019; Zhang et al., 2022). Recent studies have highlighted the potential of RUT and its derivatives to modulate the activity of the NLRP3 inflammasome and attenuate inflammation in various disease models (Luo et al., 2020). However, RUT has low oral bioavailability, which has led us to design RUT derivatives to improve the *in vivo* bioavailability while maintaining the anti-inflammation effect. 5-deoxy-rutaecarpine (5-DR), a derivative of RUT that has been enhanced for better water solubility and efficacy (Luo et al., 2020), was employed in this study. Our objectives were to determine whether 5-DR is a useful treatment for reducing LPS-induced ALI and to clarify the underlying mechanisms.

## Materials and methods

### Chemicals, reagents, and antibodies

**FIGURE 1:**
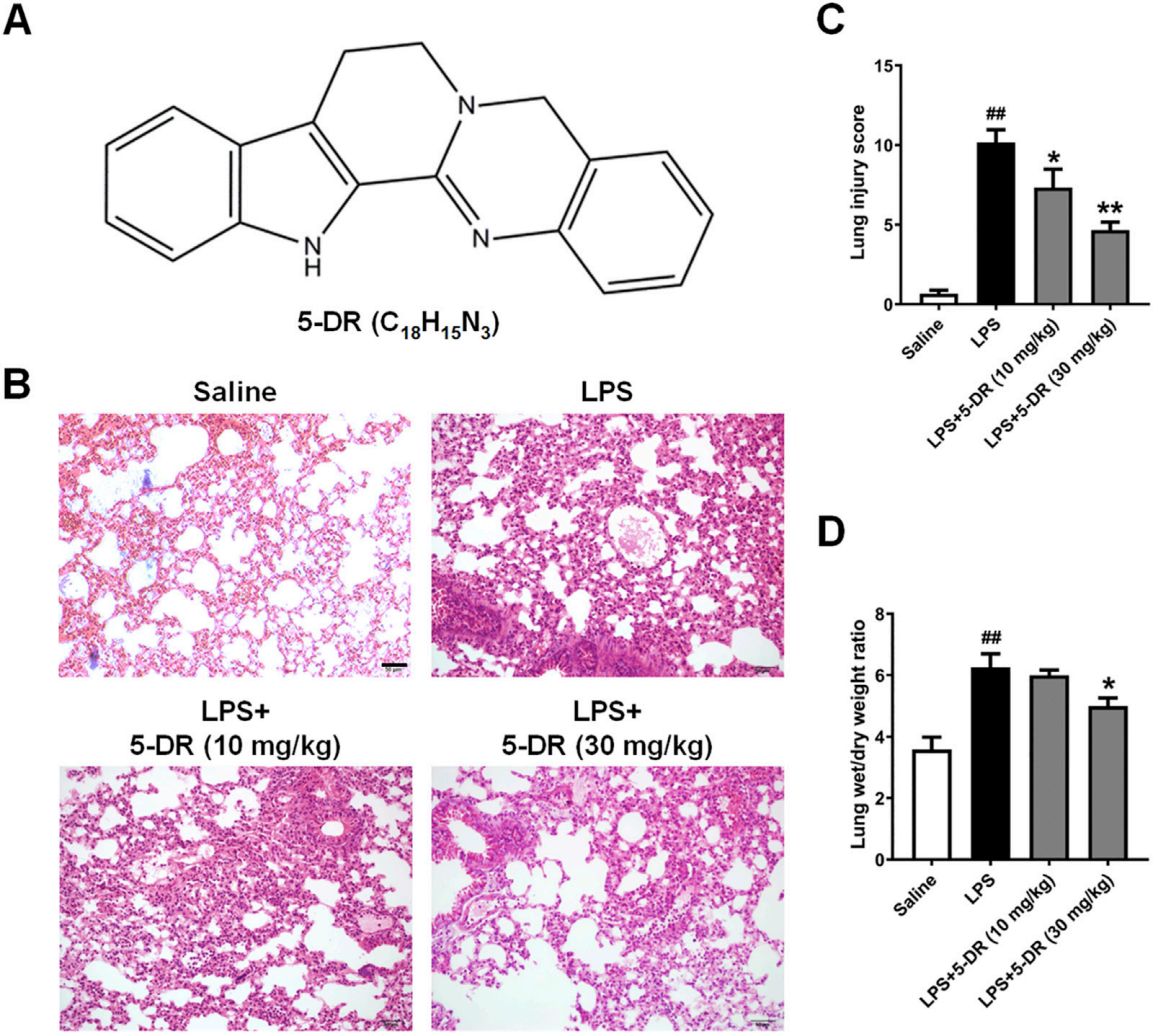
Effects of 5-DR on LPS-challenged mice. C57BL/6 mice were administered with 5-DR (10, 30 mg/kg) via gastric gavage once per day for 8 days before being treated by intratracheal instillation of LPS (5 mg/kg) or saline for 24 h and then the lung tissues were harvested. The survival rate of mice was recorded for up to 72 h. **(A)** Chemical structure of 5-DR. **(B)** Representative images of H&E-stained lung sections. Scale bar corresponds to 50 µm. **(C)** Histopathological lung injury score. Results were presented as mean ± SEM (n = 6). ^##^ *P* < 0.01 vs. Saline; *P < 0.05, **P < 0.01 vs. LPS. **(D)** Lung wet/dry weight ratio. Results were presented as mean ± SEM (n = 6). ^##^ *P* < 0.01 vs. Saline; *P < 0.05 vs. LPS. Statistical analysis was performed by one-way ANOVA plus Dunnett’s multiple comparisons test.

We synthesized 5-deoxy-rutaecarpine (5-DR) in our lab (Figure 1A). Lipopolysaccharide (*Escherichia coli* O55: B5, LPS) was purchased from Sigma Chemical Co. (St. Louis, MO, United States). RPMI-1640 cell culture medium was obtained from KeyGEN BioTECH (Nanjing, China). Fetal bovine serum (FBS) was purchased from Gibco (Waltham, MA, United States). Mouse IL-1β, IL-18, TNF-α, IL-6, and IL-10 (enzyme-linked immunosorbent assay) ELISA kits were obtained from Dakewe Biotech Co., Ltd. (Shenzhen, China). Mouse NO ELISA kit was obtained from Wuhan Saipei Biotechnology Co., Ltd. (Wuhan, China). pcDNA3.1-IKKβ plasmid was purchased from Genscript Co., Ltd. (Nanjing, China). Antibodies against p-IκBα, IκBα, p-p65, and p65 were purchased from Bioworld Technology, Inc. (Nanjing, China); Antibody against Caspase-1, Lamin B1, and GAPDH were purchased from HuaBio Biotech Co., Ltd. (Hangzhou, China). Antibody against NLRP3, ASC, IL-1β, and IKKβ was purchased from Cell Signaling Technology, Inc. (Boston, MA, United States). The other chemicals and reagents used were of analytical grade available.

### Animals and experimental protocols

Male C57/BL6 mice (6–8 weeks old) were obtained from Changsha Tianqin Biotechnology Co., Ltd. (Changsha, China). All animal experiments were conducted in accordance with the guidelines for the Care and Use of Laboratory Animals of the National Institutes of Health and the related ethical regulations of Changsha Medical University (IACUC approval no: D2023022). Mice were randomly divided into four groups: Saline group, LPS group, LPS+5-DR (10 mg/kg) group, and LPS+5-DR (30 mg/kg) group. 5-DR (dissolved in 0.5% CMC-Na) was administered once per day via gastric gavage according to the experimental design. On the 8th day, 2 h after gavage, the mice were treated by intratracheal instillation of LPS (5 mg/kg) (Luo et al., 2020; Wu et al., 2018; Qin et al., 2024; Lee C. et al., 2019). Mice in the Saline group were administered an equal volume of saline in the same schedule.

In the animal experiment, mice were anesthetized (3% isoflurane for induction, 1.5%–2% for maintenance) at 24 h post LPS stimulation, and sacrificed by cervical dislocation after blood collection from the eyeball. Mice death was confirmed by the absence of a heartbeat, lack of respiratory movement, and non-responsiveness to physical stimuli. The right lung of six mice from each group was used to calculate the lung wet/dry weight ratio, whereas the left lung was used to collect bronchoalveolar lavage fluid (BALF). The right lung of the other six mice in each group was utilized for histological study with Hematoxylin-eosin (H&E) staining, while the left lung was used for Western blotting.

### Histopathologic analysis

Lungs from mice in all groups were removed and immediately fixed in 10% formalin. The fixed samples were embedded in paraffin, sectioned, and stained with H&E (Su et al., 2023). As previously described, the stained sections were examined blindly under a microscope to compute the lung injury score (Liu et al., 2016). Briefly, we conducted a semi-quantitative analysis of lung injury in mice according to the following criteria: (1) Alveolar hemorrhage; (2) Alveolar edema; (3) Alveolar and interstitial inflammation; (4) Alveolar wall thickening and/or hyaline membrane. Briefly, the histological changes were scored on a scale of 0–4 (0, normal; 1, mild; 2, moderate; 3, severe; 4, intense). Results were confirmed by an experienced and qualified pathologist.

### Lung wet/dry weight ratio

Lungs of mice were obtained, and the wet weights were obtained immediately. Then, lungs were dried in an oven at 80°C for 48 h until achieving stable dry weights. The lung wet/dry weight ratio was calculated as the indicator of lung edema.

### Collection of BALF

The lungs were lavaged with 0.5 mL of PBS four times. The BALF was centrifuged at 1,500 rpm for 10 min at 4°C. The supernatant was collected and kept at −80°C for subsequent analysis.

### Transmission electron microscopy

Lung tissue samples were sectioned into 1–2 mm³ cubes and fixed in 2.5% glutaraldehyde buffer at 4°C for 24 h. The samples were subsequently washed in PBS, post-fixed with 1% osmium tetroxide, dehydrated using a graded ethanol series, and embedded in epoxy resin. Ultrathin sections (70 nm) stained with uranyl acetate and lead citrate were analyzed with a transmission electron microscope (JEM1400, JEOL, Japan), images of each sample were captured at ×10000 magnifications.

### Cell culture and treatment

Murine macrophage J774A.1 cells were obtained from ATCC and cultured in RPMI-1640 medium containing 10% FBS, 100 U/mL penicillin, and 100 μg/mL streptomycin at 37°C in a 5% CO_2_ humidified air.

To investigate the effect of 5-DR on cell viability, J774A.1 cells were seeded in 96-well plates and cultured in a 37°C, 5% CO_2_ incubator for 24 h. Then, they were incubated with 5-DR at different concentrations (0, 0.01, 0.1, 1, 5, 10, 25, 50, 100 μM) in the absence or presence of LPS (1 μg/mL) for 24 h (Luo et al., 2015), and the cell viability was tested by MTT assay.

To investigate the effect of 5-DR on inflammation, J774A.1 cells were seeded in 6-well plates and pretreated with different concentrations of 5-DR (0, 0.01, 0.1, 1 μM) for 20 h, then treated with LPS (1 μg/mL) for 4 h (Luo et al., 2020). The supernatants were collected for ELISA, and cell protein lysates were used for Western blotting.

For cell transfection-related assay, J774A.1 cells were seeded in 6-well plates and cultured in a 37°C, 5% CO_2_ incubator for 24 h. Then the pcDNA3.1-IKKβ-overexpressing plasmid (4 μg/well) or pcDNA3.1 plasmid (4 μg/well) was transfected into J774A.1 cells using Lipofectamine 2000 reagent (Invitrogen, CA, Carlsbad, United States) according to the manufacturer’s instructions. After 4 h of transfection, the cells were treated with or without 5-DR (1 μM) for 20 h, then treated with LPS (1 μg/mL) for 4 h. The supernatants were collected for ELISA, and cell protein lysates were used for Western blotting.

### MTT assay

Cells were cultured under indicated conditions, 20 μL of MTT solution (5 mg/mL in PBS) was added into each well and cultured for 4 h. The supernatants were removed, and the formazan crystals were dissolved in DMSO (150 µL/well). The absorbance was measured at 570 nm using a Model 1,500 Multiskan spectrum microplate Reader (Thermo, Waltham, MA, United States) (Song et al., 2023; Yao et al., 2024).

### ELISA

The concentrations of inflammatory cytokines (IL-1β, IL-18, TNF-α, IL-6, NO, and IL-10 in serum; IL-1β, IL-18, TNF-α, and IL-6 in BALF and cell supernatants) were detected by using ELISA kits according to the manufacturer’s instructions.

### Luciferase reporter assay

The NLRP3 promoter reporter vector was designed and constructed by Shanghai Sangon Biotech Co., Ltd. (Shang hai, China). The J774A.1 cells were transiently transfected with luciferase reporter plasmids, Renila control luciferase vector, pcDNA3.1-IKKβ-overexpressing plasmid or pcDNA3.1 plasmid. After 7 h of transfection, the cells were treated with or without 5-DR (1 μM) for 20 h, then treated with LPS (1 μg/mL) for 4 h. Dual Luciferase Reporter Assay System (Promega, United States) was used to measure luciferase activity (Li et al., 2024; Luo et al., 2024).

### Western blotting assay

Protein lysates from J774A.1 cells and lung homogenates were prepared following standard protocols and the proteins from the cell culture supernatants were extracted using a Liquid sample protein extraction kit (Biao Leibo, Beijing, China) (Luo et al., 2024). All protein samples were separated on 10% sodium dodecyl sulfate polyacrylamide gel electrophoresis (SDS-PAGE) and transferred to PVDF membranes (Millipore, Burlington, MA, United States). Following by blocking with 5% bovine serum albumin (BSA, Beyotime) in TBST (Tris-buffered saline, 0.1% Tween 20) for 1 h at room temperature, the membranes were incubated with different antibodies overnight at 4°C. Membranes were washed three times and incubated with secondary antibodies for 1 h at room temperature. Protein bands were detected with an Ultra-sensitive ECL chemiluminescence kit (Beyotime) on a chemiluminescence imaging system (Tanon 5,200, Yuanpinghao Biotechnology, Beijing, China) and analyzed using ImageJ software. The dilutions of both primary and secondary antibodies were listed in Supplementary Table S1.

### Statistical analysis

Data are presented as the means ± SEM. Statistical analysis was performed with one-way analysis of variance (ANOVA) using GraphPad Prism. For the results of survival experiments, the log-rank test was used. *P* values less than 0.05 (*P* < 0.05) were considered statistically significant.

## Results

### Effects of 5-DR on LPS-induced ALI in mice

To evaluate the impact of 5-DR on LPS-induced lung injury, the histological alterations in lung tissue were examined using H&E staining. As shown in Figure 1B, Histological examination of lung tissues from mice subjected to ALI induction by LPS revealed significant histopathological alterations characteristic of lung injury. These changes included alveolar congestion, hemorrhage, and infiltration of inflammatory cells, indicating the development of ALI. However, treatment with 5-DR (10, 30 mg/kg) mitigated these histopathological changes, preserving lung architecture and reducing inflammatory cell infiltration. Quantitative analysis of lung injury score also demonstrated treatment with 5-DR significantly attenuated lung injury (Figure 1C), Lung sections from mice treated with 5-DR exhibited decreased lung inflammation and alveolar wall destruction compared to the untreated ALI group. Calculating the wet-to-dry weight ratio of the lung showed that LPS-induced ALI increased lung water content, indicating pulmonary edema. However, treatment with 5-DR attenuated lung edema, as evidenced by a significant reduction in the wet-to-dry weight ratio compared to the untreated ALI group (Figure 1D). In addition, transmission electron microscopy was used to examine the ultrastructural changes of lung tissues (Supplementary Figure S1). In the LPS group, cytoplasmic vacuolation, disorganized and fragmented mitochondrial cristae, marked mitochondrial swelling and vacuolation were observed, indicating cellular stress and injury. When compared to the LPS group, the pathologic damage was significantly alleviated in the LPS+5-DR (30 mg/kg) group, with reduced cytoplasmic vacuolation, more integrated and manifested of morphology, reduced disorganization of mitochondrial cristae, and less swelling. These results demonstrated the protective effect of 5-DR against LPS-induced lung damage.

### Effects of 5-DR on the levels of inflammatory cytokines in LPS-induced ALI in mice

**FIGURE 2:**
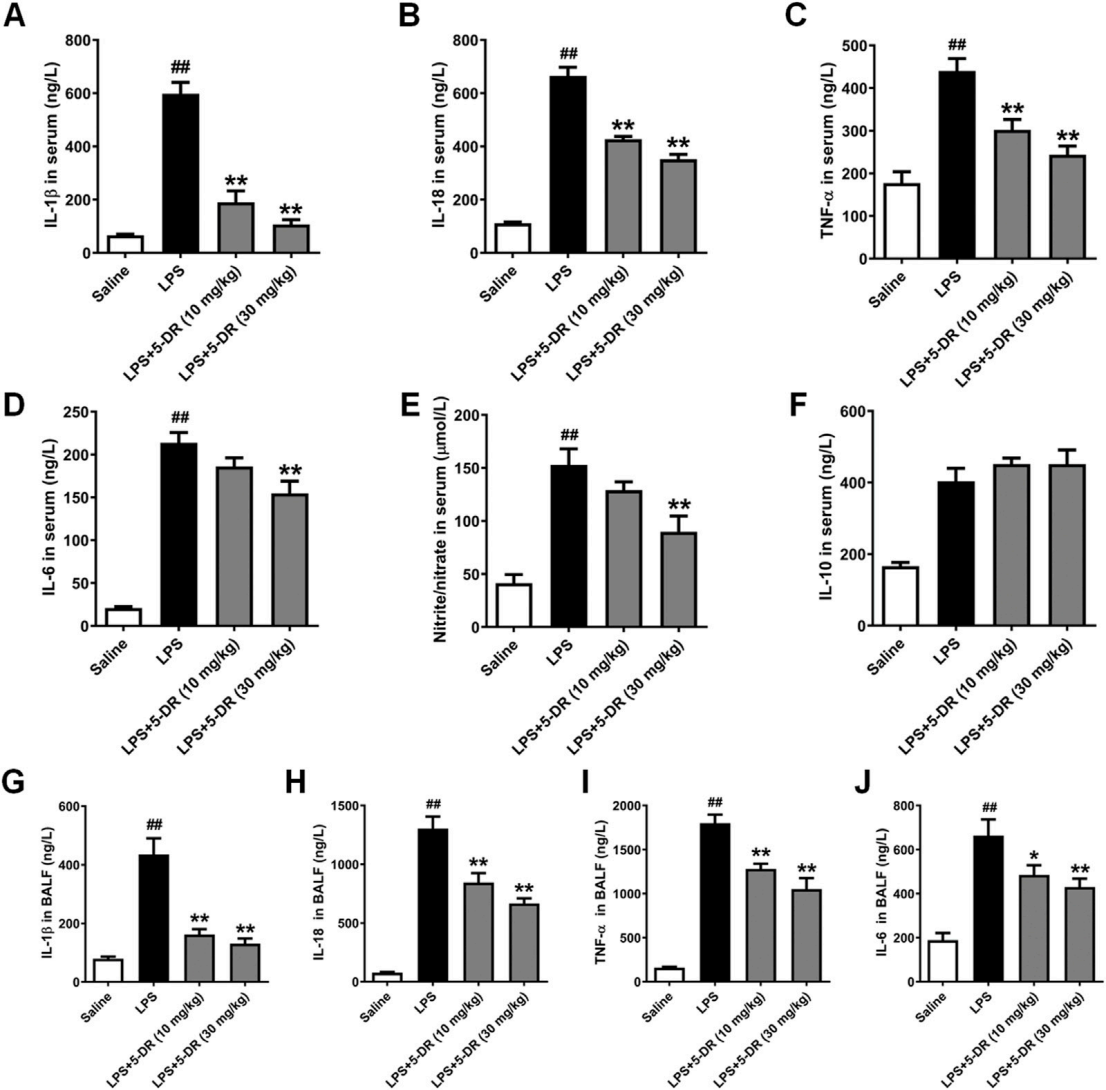
Effects of 5-DR on the levels of inflammatory cytokines in LPS-challenged mice. C57BL/6 mice were administered with 5-DR (10, 30 mg/kg) via gastric gavage once per day for 8 days before being treated by intratracheal instillation of LPS (5 mg/kg) or saline for 24 h and then the BALF and serum were collected. **(A–F)** Levels of IL-1β, IL-18, TNF-α, IL-6, nitrite/nitrate, and IL-10 in serum were determined by ELISA kits. **(G–J)** Levels of IL-1β, IL-18, TNF-α, and IL-6 level in BALF. Results were presented as mean ± SEM (n = 6). ^##^ *P* < 0.01 vs. Saline; *P < 0.05, **P < 0.01 vs. LPS. Statistical analysis was performed by one-way ANOVA plus Dunnett’s multiple comparisons test.

Inflammatory cytokines play a critical role in the occurrence and development of LPS-induced ALI (Kan et al., 2021; Feng et al., 2022; Liu et al., 2023). Understanding the effects of 5-DR on inflammatory cytokines in LPS-induced ALI is essential for elucidating its therapeutic potential in this condition. The results showed that 5-DR administration significantly suppressed the serum levels of pro-inflammatory cytokines IL-1β, IL-18, TNF-α, Nitrite/nitrate, and IL-6 in LPS-intoxicated mice, although had no significant effect on IL-10 level (Figures 2A–F). We further examined the effect of 5-DR on the BALF levels of IL-1β, IL-18, TNF-α, and IL-6. Results show that the BALF levels of IL-1β, IL-18, TNF-α, and IL-6 (Figures 2G–J) were significantly upregulated in response to LPS, while 5-DR (10, 30 mg/kg) administration could reduce inflammatory cytokine levels caused by LPS. By attenuating the production of inflammatory cytokines, 5-DR can effectively modulate the inflammatory response, thereby protecting lung tissue from excessive inflammation and damage.

### Effects of 5-DR on the expressions of inflammatory cytokines in LPS-stimulated J774A.1 cells

**FIGURE 3:**
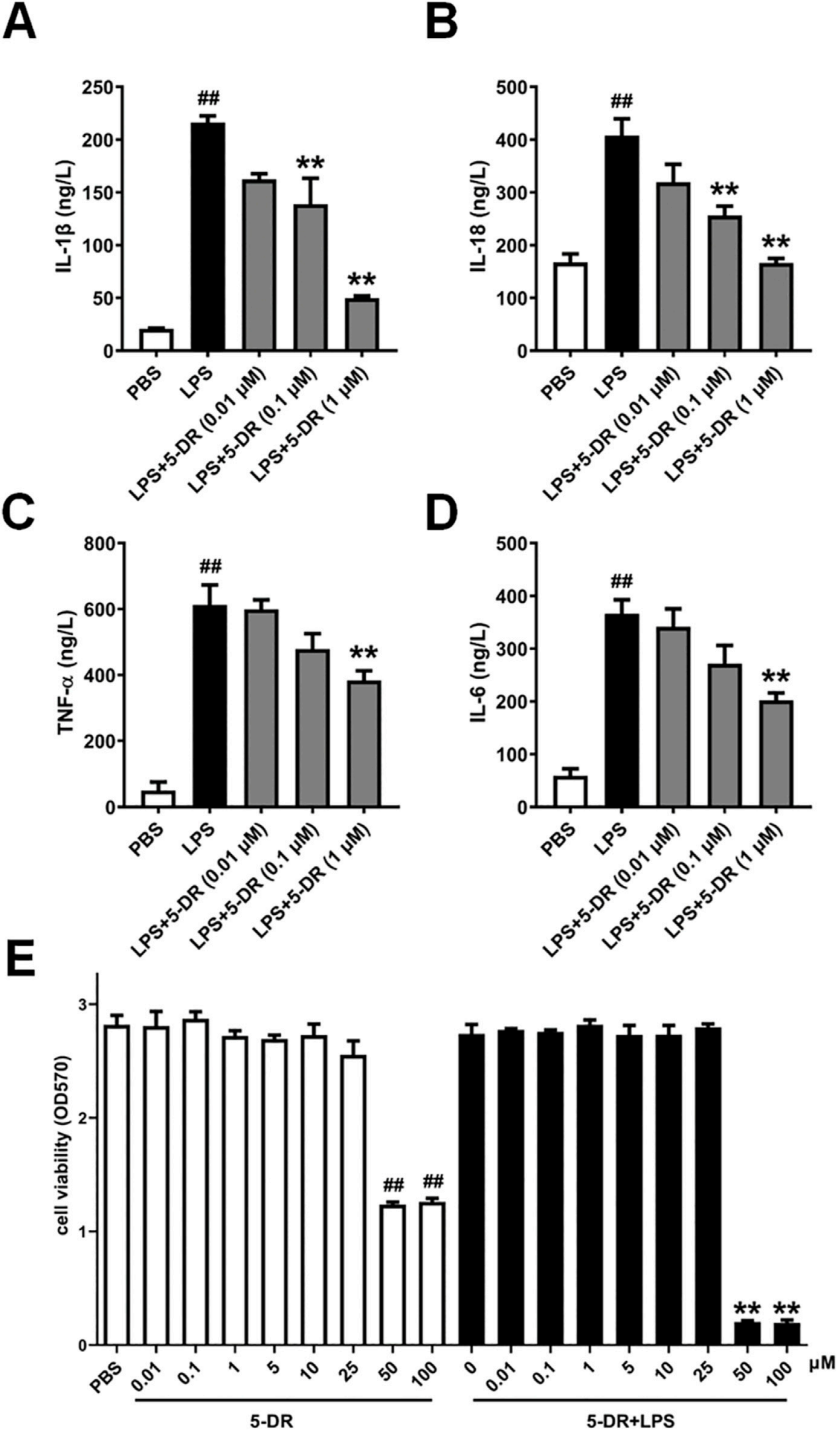
Effects of 5-DR on the expressions of inflammatory cytokines in LPS-stimulated J774A.1 cells. **(A–D)** J774A.1 cells were treated with LPS (1 μg/mL) for 4 h, after pretreated with different concentrations of 5-DR (0.01, 0.1, 1 μM), and the culture supernatants were collected. IL-1β, IL-18, TNF-α, and IL-6 levels were determined by ELISA kits. **(E)** J774A.1 cells were treated with 5-DR (0.01, 0.1, 1, 5, 10, 25, 50, 100 μM) in the presence or absence of LPS (1 μg/mL) for 24 h, cell viability was determined by using MTT assay. Results were presented as mean ± SEM (n = 6). ^##^ *P* < 0.01 vs. PBS; **P < 0.01 vs. LPS. Statistical analysis was performed by one-way ANOVA plus Dunnett’s multiple comparisons test.

Macrophages are the primary producers of proinflammatory/anti-inflammatory cytokines such as IL-1β, IL-18, TNF-α, and IL-6, which play a significant role in controlling the inflammatory process (Sun et al., 2019; Al-Qahtani et al., 2024; Li et al., 2023). We then evaluated the effects of 5-DR on cytokine expressions in LPS-stimulated J774A.1 macrophages, a murine macrophage cell line commonly used to study inflammation. Treatment with LPS significantly upregulated the expressions of IL-1β, IL-18, TNF-α, and IL-6 in J774A.1 cells compared to PBS-treated controls, consistent with the pro-inflammatory response induced by LPS stimulation *in vivo*. While pre-treatment with 5-DR (0.01, 0.1, 1 μM) dose-dependently attenuated the LPS-induced increase in inflammatory cytokine expressions (Figures 3A–D).

To exclude the possibility that the anti-inflammation effect of the test compounds on the J774A.1 cells was due to cytotoxicity, effects of 5-DR on the viability of J774A.1 cells in the presence or absence of LPS (1 μg/mL) was detected using an MTT assay. The results showed that although when LPS and 5-DR are used together, cells are more susceptible to additional stressors due to the signaling pathways activated by LPS (Yuan et al., 2009), 5-DR remains not affect the viability of J774A.1 cells at concentrations below 25 μM (Figure 3E).

### Effects of 5-DR on the NLRP3 inflammasome activation in LPS-induced ALI in mice

**FIGURE 4:**
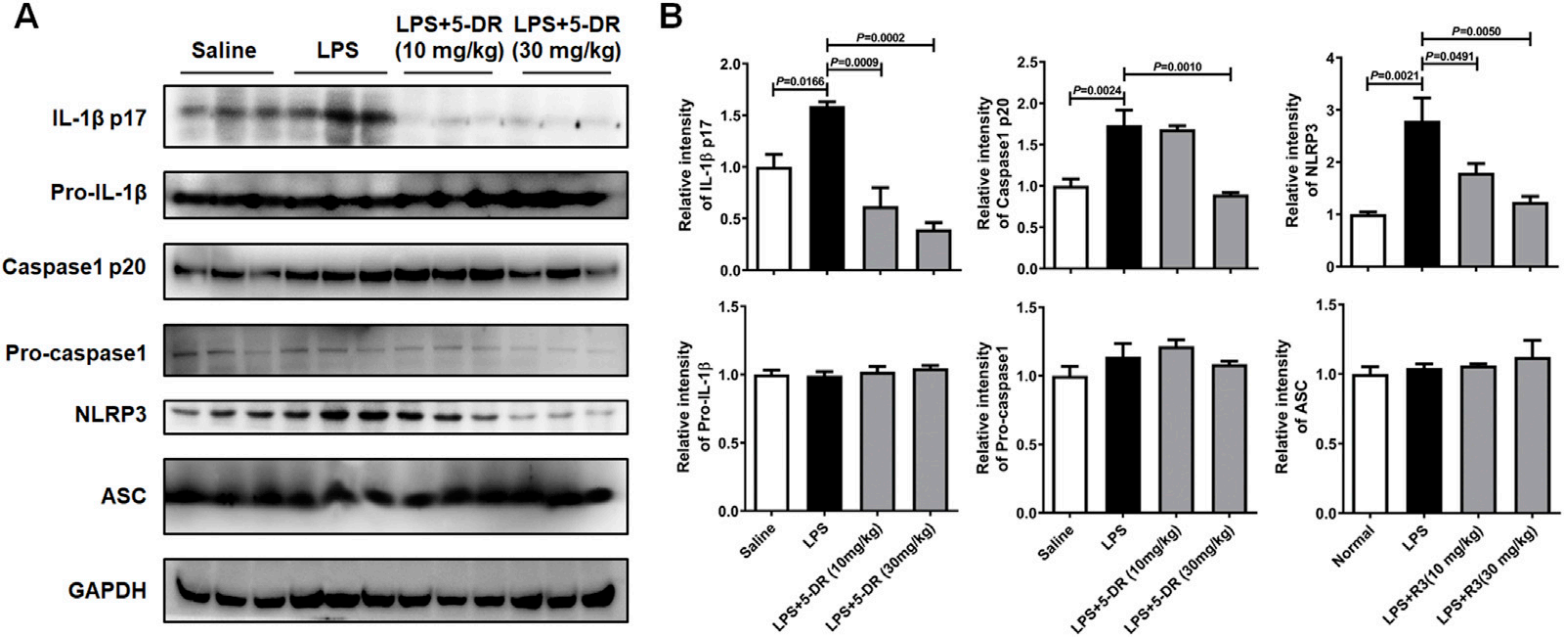
Effects of 5-DR on the NLRP3 inflammasome activation in LPS-challenged mice. **(A)** Western blotting analysis of IL-1β p17, pro-IL-1β, caspase-1 p20, pro-caspase-1, NLRP3, ASC, and GAPDH in mouse lung tissue exposed to LPS with or without 5-DR treatments. **(B)** Quantitative analysis of IL-1β p17, pro-IL-1β, caspase-1 p20, pro-caspase-1, NLRP3, and ASC protein levels. Results were presented as mean ± SEM (n = 3). Statistical analysis was performed by one-way ANOVA plus Dunnett’s multiple comparisons test.

The NLRP3 inflammasome is a multiprotein complex that plays a crucial role in the innate immune system by initiating an inflammatory response (Yao et al., 2024). It is activated in response to various danger signals, including microbial products like LPS, as well as endogenous danger signals associated with tissue damage or stress. The role of NLRP3-related inflammation in ALI has been well identified previously (Wang and Zhao, 2022; Liu et al., 2021). In our study, 5-DR treatment markedly suppressed the levels of IL-1β and IL-18 both in BALF and serum of LPS-challenged mice, which are the products of NLRP3 inflammasome activation (Figure 3). Then, we examined whether the anti-inflammatory role of 5-DR was related to the NLRP3 inflammasome. When activated, the NLRP3 inflammasome recruits the adaptor protein ASC (apoptosis-associated speck-like protein containing a CARD) (Proell et al., 2013), which acts as a bridge to recruit pro-caspase-1, leading to its proximity-induced self-cleavage and activation. Caspase-1 is initially synthesized as an inactive zymogen (pro-caspase-1). Upon activation, it is cleaved into two subunits: p20 and p10 (Bolívar et al., 2019). Activated caspase-1 then processes pro-inflammatory cytokines such as IL-1β and IL-18 into their mature forms, which are secreted by the cell to propagate the inflammatory response (Zheng et al., 2020). Western blotting analysis revealed that ALI induction by LPS resulted in increased expression levels of NLRP3, cleaved caspase-1 p20, and mature IL-1β in lung tissues, indicating activation of the NLRP3 inflammasome and subsequent release of pro-inflammatory cytokines (Figures 4A, B). However, treatment with 5-DR significantly inhibited NLRP3 inflammasome activation, as evidenced by decreased expression levels of NLRP3, cleaved caspase-1 p20, and mature IL-1β in lung tissues compared to the LPS group. The above findings are sufficient to show that 5-DR suppressed NLRP3 inflammasome-related inflammation in ALI mice, thereby attenuating pulmonary inflammation and tissue damage.

### Effects of 5-DR on the NLRP3 inflammasome activation in LPS-stimulated J774A.1 cells

**FIGURE 5:**
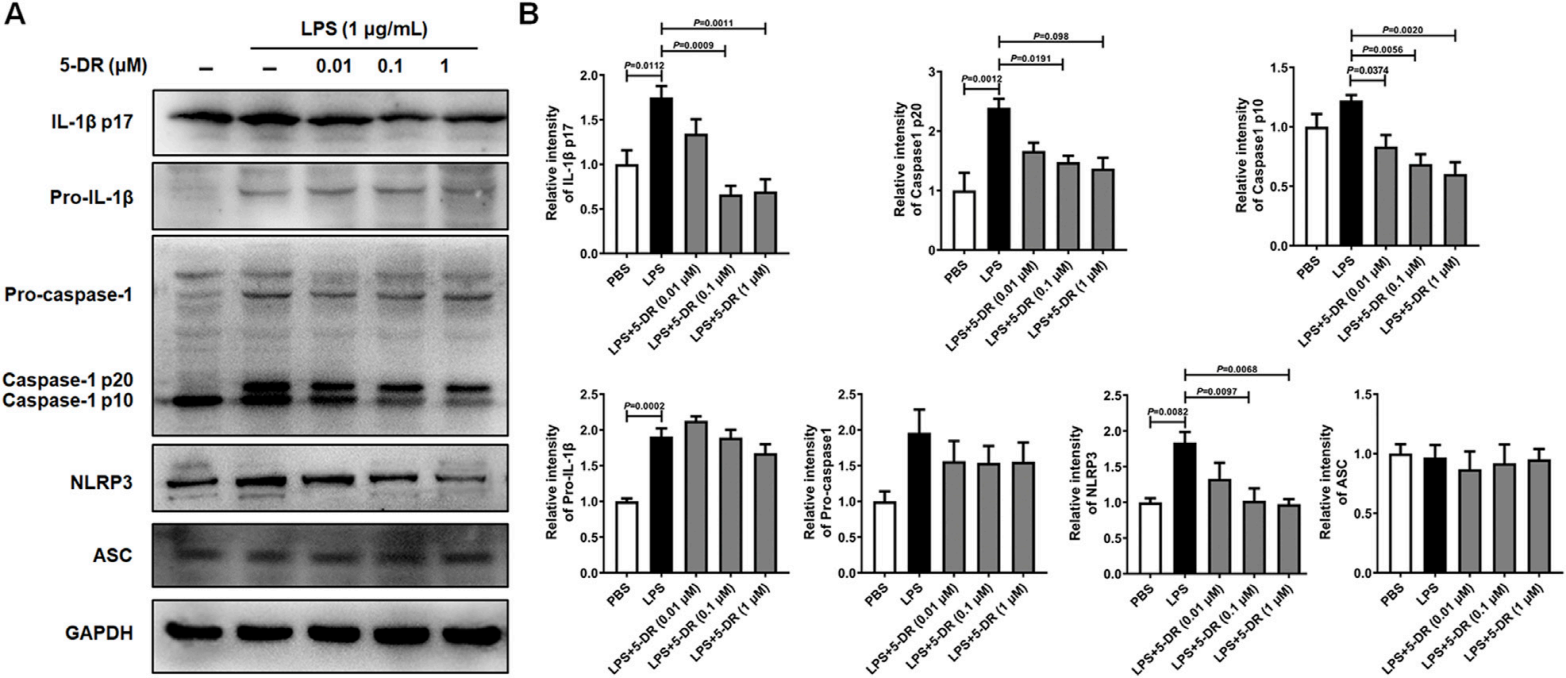
Effects of 5-DR on the NLRP3 inflammasome activation in LPS-stimulated J774A.1 cells. J774A.1 cells were pre-incubated with different concentrations of 5-DR (0.01, 0.1, 1 μM) for 20 h, then stimulated with LPS (1 μg/mL) for 4 h. Cell lysates were collected. **(A)** IL-1β p17, pro-IL-1β, caspase-1 p20, caspase-1 p10, pro-caspase-1, NLRP3, ASC, and GAPDH expression were measured by Western blotting analysis. **(B)** Quantitative analysis of IL-1β p17, pro-IL-1β, caspase-1 p20, caspase-1 p10, pro-caspase-1, and NLRP3 protein levels. Results were presented as mean ± SEM (n = 3). Statistical analysis was performed by one-way ANOVA plus Dunnett’s multiple comparisons test.

It is widely accepted that LPS stimulation triggers the activation of the NLRP3 inflammasome in macrophages (Gurung et al., 2015; Zhang et al., 2020). We next investigated whether 5-DR directly inhibited NLRP3 inflammasome activation in LPS-stimulated J774A.1 murine macrophages. Treatment with LPS significantly induced the activation of the NLRP3 inflammasome in J774A.1 cells, as evidenced by increased cleavage of caspase-1 and maturation of IL-1β compared to untreated controls. However, pre-treatment with 5-DR decreased the cleavage of IL-1β by LPS in a dose-dependent manner, and decreased the levels of NLRP3, cleaved caspase-1 p10, and cleaved caspase-1 p20 (Figures 5A, B). Overall, these results demonstrated that 5-DR effectively suppressed the activation of the NLRP3 inflammasome in LPS-stimulated macrophages.

### 5-DR treatment inhibited NLRP3 inflammasome activation through nuclear factor kappa B signaling

**FIGURE 6:**
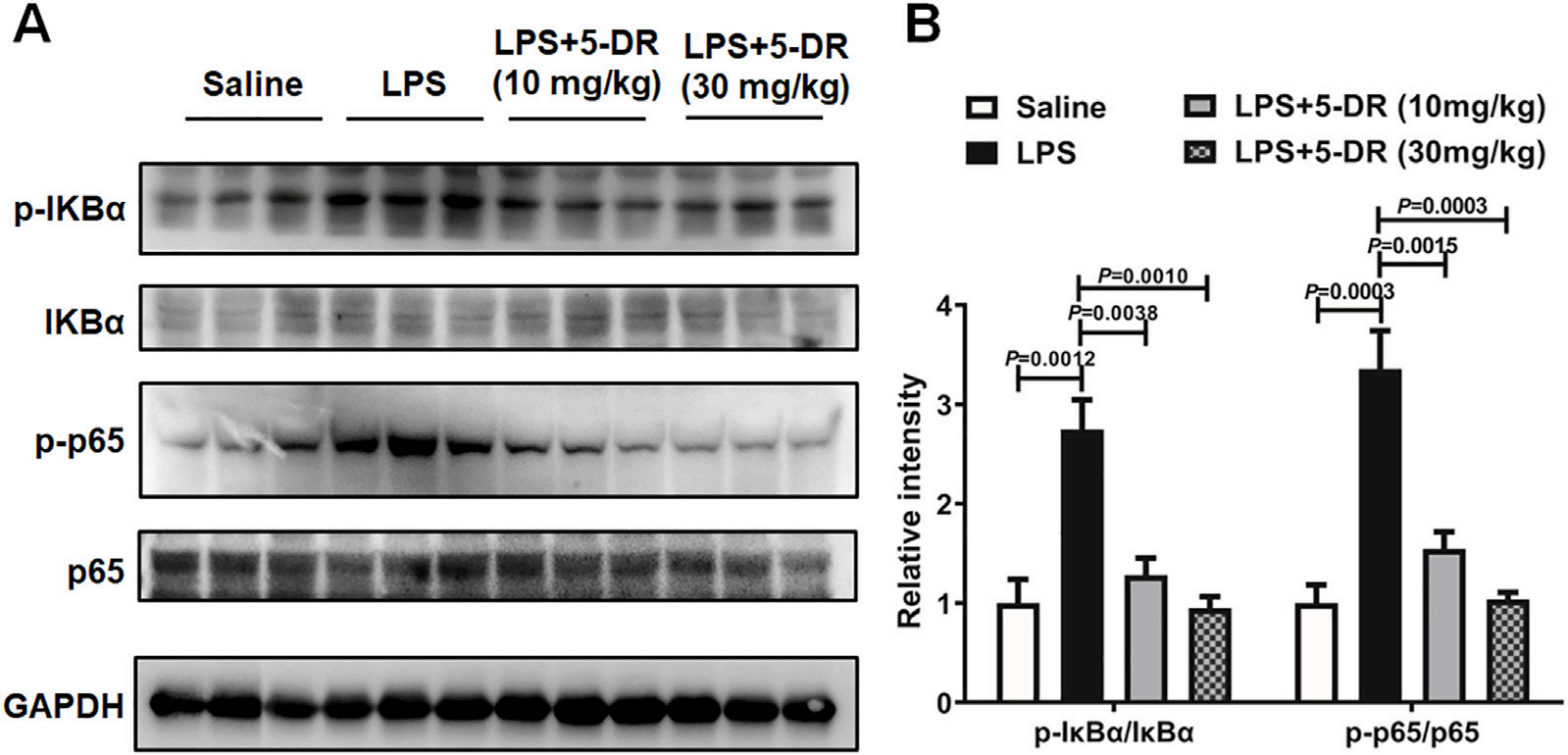
5-DR treatment inhibited NF-κB signaling in the lung of LPS-challenged mice. **(A)** Western blotting was performed to evaluate the protein expression of p-IκBα, IκBα, p-p65, p65, and GAPDH in lung lysates of mice treated with or without 5-DR. **(B)** Quantitative analysis of indicated protein levels in **(A)**. Results were presented as mean ± SEM (n = 3). Statistical analysis was performed by one-way ANOVA plus Dunnett’s multiple comparisons test.

In the context of LPS-triggered NLRP3 inflammasome activation, nuclear factor kappa B (NF-κB) serves as a crucial priming signal (Kelley et al., 2019). NF-κB activation leads to the transcriptional upregulation of NLRP3, pro-IL-1β, and pro-IL-18, thereby priming the cell for subsequent inflammasome activation (Xu et al., 2020; Yu and Cheng, 2023). Understanding the intricate crosstalk between NF-κB signaling and the NLRP3 inflammasome pathway is essential for unraveling the mechanisms of 5-DR. We then investigated if the anti-inflammatory properties of 5-DR relied on its capacity to block NF-κB by evaluating the impact of 5-DR on NF-κB signaling. Western blotting research showed that ALI induction by LPS resulted in heightened NF-κB activation in lung tissues, demonstrated by higher levels of phosphorylated NF-κB p65 (p-p65) and phosphorylated IκBα (p-IκBα) subunit. Treatment with 5-DR inhibited NF-κB activation by reducing p-p65 and p-IκBα levels relative to the untreated ALI group (Figure 6). 5-DR treatment also markedly inhibited the LPS-induced phosphorylation of IκBα and the p65 subunit in J774A.1 cells, which indicated that 5-DR may inhibit NF-κB signaling (Supplementary Figure S2). In addition, 5-DR significantly inhibited the translocation of p65 subunit from the cytoplasm to the nucleus, potentially explaining the inactivation of NF-κB signaling by 5-DR.

**FIGURE 7:**
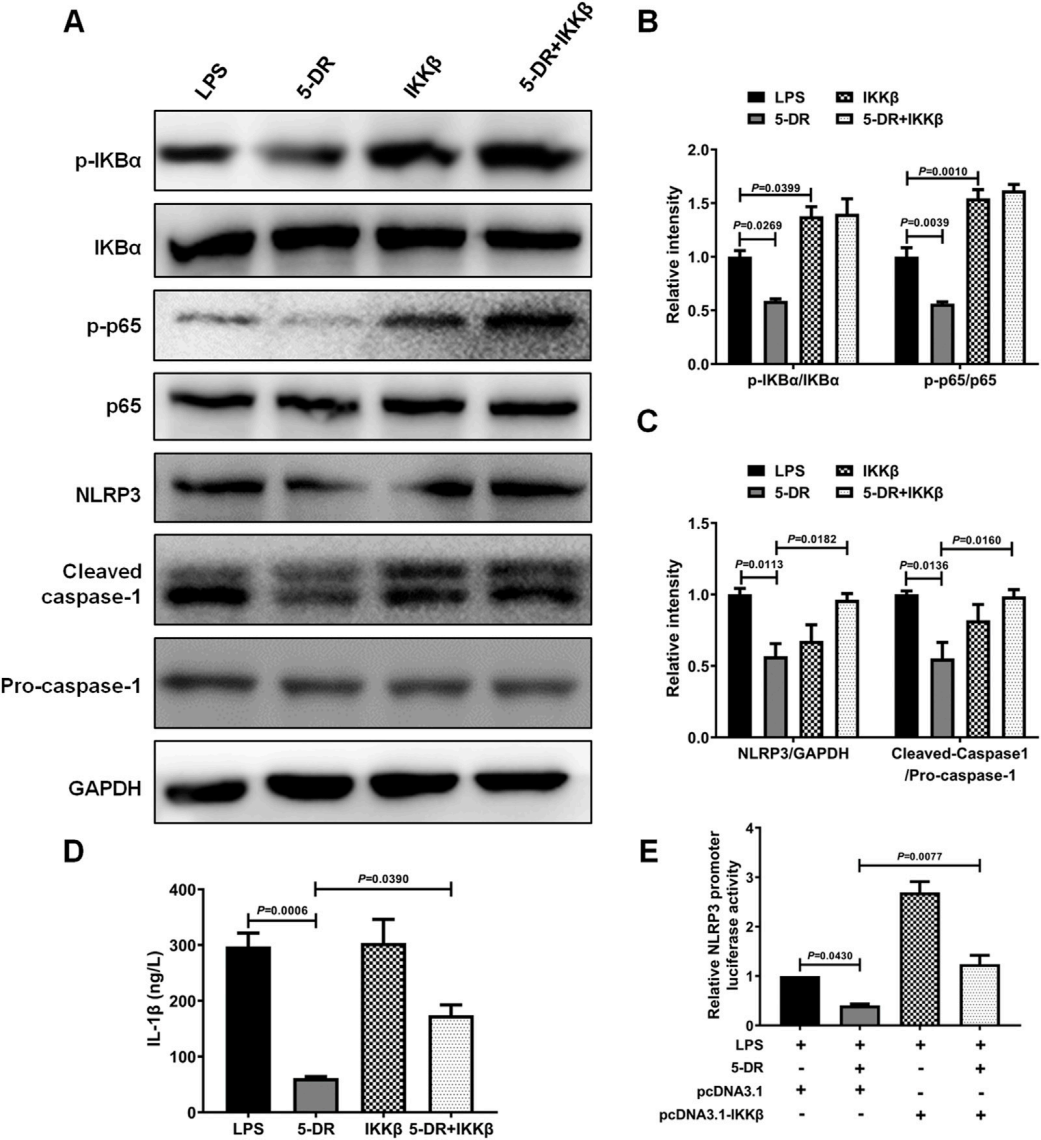
5-DR inhibited NLRP3 inflammasome activation through inhibiting NF-κB signaling. **(A–C)**, IκBα, p-IκBα, p65, p-p65, NLRP3, cleaved-caspase-1, pro-caspase-1 protein levels were measured by Western blotting, and quantified. **(D)** IL-1β in the cell supernatants was detected by ELISA. **(E)** NLRP3 promoter activity in the indicated groups was measured using luciferase assay. Results were presented as mean ± SEM (n = 3). Statistical analysis was performed by one-way ANOVA plus Dunnett’s multiple comparisons test.

The impact of NF-κB signaling on the suppression of 5-DR-mediated NLRP3 inflammasome was assessed in LPS-stimulated J774A.1 cells with and without activation of NF-κB signaling. J774A.1 cells were transfected with pcDNA3.1-IKKβ to simulate the activation of the NF-κB pathway, followed by treatment with LPS. Overexpression of IKKβ significantly increased the levels of p-IκBα and p-p65, indicating activation of the NF-κB signaling pathway (Supplementary Figure S3; Figures 7A, B). 5-DR treatment significantly reduced LPS-induced IκBα and p65 phosphorylation, attenuated NLRP3 inflammasome activation, and reduced IL-1β secretion in J774A.1 cells, while overexpression of IKKβ reversed the downregulation of NLRP3, cleavage of caspase-1, and decrease in IL-1β induced by 5-DR (Figures 7A–C). Subsequently, to explore whether 5-DR could regulate NLRP3 promoter activity in J774A.1 cells, we transfected J774A.1 cells with a luciferase reporter plasmid containing NLRP3 promoter region. Our results demonstrated that 5-DR treatment decreased NLRP3 promoter activity. However, overexpression of IKKβ could diminish the inhibition of NLRP3 promoter activity caused by 5-DR (Figure 7E). In summary, these findings showed that 5-DR inhibit NLRP3 though the upstream NF-κB pathway and then transcriptionally decreased the activity of the NLRP3 promoter.

## Discussion

ALI/ARDS is characterized by excessive lung inflammation and tissue damage, often resulting in significant morbidity and mortality (Li G. X. et al., 2022; Qiu et al., 2018). Despite advances in supportive care strategies such as mechanical ventilation and fluid management, there remains a critical need for targeted pharmacological interventions to mitigate inflammation, preserve lung function, and improve outcomes in patients with ALI/ARDS (Liaqat et al., 2022). Therefore, developing new drugs or treatment strategies for ALI/ARDS is crucial for improving patient outcomes, and addressing unmet medical needs. In the present study, we demonstrated that 5-DR inhibits NLRP3 inflammasome-related inflammation, thereby identifying it as a potential therapeutic drug for ALI.

RUT is a bioactive compound with diverse pharmacological properties, including vasodilatory, anti-inflammatory, antioxidant, and cardioprotective effects (Ko et al., 2007; Heo et al., 2009; Xu et al., 2014; Liao et al., 2021). However, its poor physicochemical properties and moderate biological activities have hampered its clinical application. 5-DR is a derivative of RUT with higher solubility and efficacy than RUT. Our previous research showed that 5-DR suppressed atherosclerosis via inhibiting NLRP3 inflammasome-related inflammation and modulating cholesterol transport (Luo et al., 2020). However, its potential for use in the treatment of ALI remains to be elucidated. To date, the NLRP3 inflammasome has emerged as a critical player in the pathogenesis of ALI (Leszczyńska et al., 2022). By inhibiting NLRP3 inflammasome activation or downstream effector molecules, it may be possible to dampen the inflammatory response and attenuate lung injury (Grailer et al., 2014). Several pharmacological agents and natural compounds have been shown to modulate NLRP3 inflammasome activation and hold promise as potential therapeutics for ALI (Ozaki et al., 2015; Das et al., 2021; Wang et al., 2022). In this study, we investigated the effects of 5-DR on NLRP3 inflammasome activation and its potential therapeutic efficacy in ALI. Our results demonstrated that 5-DR effectively inhibited NLRP3 inflammasome activation in LPS-induced lung injury in mice, as evidenced by reduced expression and cleavage of NLRP3, caspase-1, and IL-1β. Combined with previous research, we speculated that 5-DR acts as a small-molecule inhibitor of the NLRP3 inflammasome and holds promise as a therapeutic agent for the treatment of ALI.

ALI often begins with an initial insult, such as infection, aspiration, or trauma, that triggers an inflammatory response in the lungs. Macrophages, particularly alveolar macrophages resident in the lung alveoli, serve as sentinel cells that recognize and respond to microbial products, such as LPS from Gram-negative bacteria, through pattern recognition receptors like Toll-like receptor 4 (TLR4) (Niesler et al., 2014; Mazgaeen and Gurung, 2020). The initial recognition of LPS by TLR4 results in the priming of the NLRP3 inflammasome (Chang et al., 2020; Ciesielska et al., 2021). This priming step involves the transcriptional upregulation of NLRP3, pro-IL-1β, and pro-IL-18 via NF-κB activation (Lawrence, 2009; Mezzasoma et al., 2023). NF-κB is a well-established upstream regulator of the NLRP3 inflammasome, acting as a transcription factor that promotes the expression of key inflammasome components, including NLRP3 and pro-IL-1β. Upon activation by various stimuli, NF-κB translocated to the nucleus and binds to specific promoter regions, driving the transcriptional upregulation of genes involved in NLRP3 inflammasome activation (Kelley et al., 2019; Qiao et al., 2012). Ongoing research into specific macrophage-targeted therapies, including cytokine inhibitors, and NLRP3 inflammasome inhibitors, holds the potential to improve outcomes for patients suffering from ALI. Consistent with previous studies, our results demonstrated that LPS administration induced a robust inflammatory response in macrophages and mice, characterized by elevated production of pro-inflammatory cytokines, activation of inflammatory signaling pathways, and tissue damage. In our study, J774A.1 macrophage line instead of RAW 264.7 macrophage line was chosen as an *in vitro* model due to the lack of ASC in RAW 264.7 cells (Zito et al., 2020; Pelegrin et al., 2008), which results in defective activation of the NLRP3 inflammasome. We discovered that 5-DR efficiently reduced the production of pro-inflammatory cytokines and blocked the NF-κB pathway activation in both J774A.1 cells and mouse model of ALI. In our investigation, the reduced expression of inflammatory cytokines such as IL-6 and TNF-α can be attributed to the suppression of NF-κB pathways with 5-DR therapy. The NF-κB pathway is acknowledged as the primary signal for the NLRP3 inflammasome in macrophages activated by LPS. NF-κB translocates to the nucleus and binds to specific promoter regions, driving the transcriptional upregulation of genes involved in NLRP3 inflammasome activation. Our data showed that the activation of the NF-κB pathway reduced the ability of 5-DR to suppress the NLRP3 inflammasome and NLRP3 promoter activity, suggesting that 5-DR may prevent NLRP3 inflammasome activation by blocking the NF-κB pathway.

The primary considerations for selecting clinical drugs are a substantial therapeutic impact and minimal adverse effects. Typically, higher medication concentrations result in increased treatment effects and adverse effects, thus limiting the utility of many treatments. Several molecular targets and agents have been proposed to treat ALI. For instance, Glycyrrhizin and Cirsilineol have shown potential in ameliorating LPS-induced ALI (Lee S. A. et al., 2019; Ai et al., 2021). However, the development of therapeutic strategies to improve ALI treatment outcomes remains limited, often due to issues with efficacy, safety, or both. In our study, we demonstrated that 5-DR at doses of 10 mg/kg and 30 mg/kg mitigate LPS-induced inflammatory responses without inducing overt toxicity. The selected dose is based on previously reported effective doses of rutaecarpine-derived compounds in animal models (Luo et al., 2020; Qin et al., 2024; Lee C. et al., 2019), suggesting a broad applicability of this dosing range. Among the tested doses, 30 mg/kg was identified as the optimal dose, producing significant anti-inflammatory effects as evidenced by reduced levels of pro-inflammatory cytokines (IL-1β, IL-18, TNF-α, IL-6) and histological improvements in the lung, while no signs of toxicity were observed. 5-DR at 30 mg/kg also significantly inhibited the activation of the NLRP3 inflammasome and relative NF-κB signaling pathway, both of which are critical mediators of inflammation (Grailer et al., 2014; Gu et al., 2024). These findings suggest that 5-DR, a novel NLRP3 inflammasome inhibitor, can mitigate inflammation in LPS-induced ALI and holds promise as a potential therapeutic agent for reducing ALI in clinical practice. The dose used in this study provides a foundation for future research aimed at translating these findings to clinical settings. However, further pharmacokinetic and toxicological studies are needed to establish the optimal therapeutic dose in humans and to evaluate long-term safety.

Our study also has several limitations. While it demonstrated significant protective effects of 5-DR against LPS-induced lung injury, the sample size was relatively small, which may have limited our ability to detect broader variability in individual responses. Moreover, the short duration of the experimental timeline may not adequately reflect the long-term efficacy or potential adverse effects of 5-DR. Although the results are promising in a murine model, critical factors such as the pharmacokinetics, bioavailability, and safety profile of 5-DR in humans remain unexplored, which are essential considerations for clinical application. Therefore, we will prioritize further validation through large animal models and subsequent preclinical studies to establish the efficacy and safety of 5-DR in future research.

## Conclusion

In conclusion, this study elucidated the potential therapeutic effects of 5-DR in mitigating LPS-induced ALI, and demonstrated that the NF-κB/NLRP3 pathway is involved in the anti-inflammatory and anti-ALI properties of 5-DR. The findings offer significant insights into the anti-inflammatory properties of 5-DR and underscore its potential as a therapeutic agent for treating inflammatory lung diseases, ultimately aiming to enhance patient outcomes and decrease the morbidity and mortality associated with these conditions.

## Data Availability

The original contributions presented in the study are included in the article/Supplementary Material, further inquiries can be directed to the corresponding authors.

## Abbreviations

5-DR, 5-deoxy-rutaecarpine; RUT, Rutaecarpine; ALI, Acute lung injury; LPS, Lipopolysaccharide; ARDS, Acute respiratory distress syndrome; NLRP3, NOD-like receptor family, pyrin domain-containing 3; PAMPs, Pathogen-associated molecular patterns; DAMPs, Damage-associated molecular patterns; FBS, Fetal bovine serum; BALF, Bronchoalveolar lavage fluid; SDS-PAGE, Sodium dodecyl sulfate polyacrylamide gel electrophoresis; ASC, Apoptosis-associated speck-like protein containing a CARD; NF-κB, Nuclear factor kappa B; TLR4, Toll-like receptor 4.

## References

[B1] Ai M. Lin S. Zhang M. Wu T. Yang N. Li Y. (2021). Cirsilineol attenuates LPS-induced inflammation in both *in vivo* and *in vitro* models via inhibiting TLR-4/NFkB/IKK signaling pathway. J. Biochem. Mol. Toxicol. 35, e22799. 10.1002/jbt.22799 33949057

[B2] Ajibowo A. O. Kolawole O. A. Sadia H. Amedu O. S. Chaudhry H. A. Hussaini H. (2022). A comprehensive review of the management of acute respiratory distress syndrome. Cureus 14, e30669. 10.7759/cureus.30669 36439591 PMC9686454

[B3] Al-Qahtani A. A. Alhamlan F. S. Al-Qahtani A. A. (2024). Pro-inflammatory and anti-inflammatory interleukins in infectious diseases: a comprehensive review. Trop. Med. Infect. Dis. 9, 13. 10.3390/tropicalmed9010013 38251210 PMC10818686

[B4] Bolívar B. E. Vogel T. P. Bouchier-Hayes L. (2019). Inflammatory caspase regulation: maintaining balance between inflammation and cell death in health and disease. FEBS J. 286, 2628–2644. 10.1111/febs.14926 31090171 PMC7065599

[B5] Chang Y. Y. Jean W. H. Lu C. W. Shieh J. S. Chen M. L. Lin T. Y. (2020). Nicardipine inhibits priming of the NLRP3 inflammasome via suppressing LPS-induced TLR4 expression. Inflammation 43, 1375–1386. 10.1007/s10753-020-01215-y 32239395

[B6] Chen Y. Xiao L. Sun G. Li M. Yang H. Ming Z. (2023). TMBIM4 deficiency facilitates NLRP3 inflammasome activation-induced pyroptosis of trophoblasts: a potential pathogenesis of preeclampsia. Biology 12, 208. 10.3390/biology12020208 36829486 PMC9953300

[B7] Ciesielska A. Matyjek M. Kwiatkowska K. (2021). TLR4 and CD14 trafficking and its influence on LPS-induced pro-inflammatory signaling. Cell. Mol. life Sci. CMLS 78, 1233–1261. 10.1007/s00018-020-03656-y 33057840 PMC7904555

[B8] Cui Y. Wang X. Lin F. Li W. Zhao Y. Zhu F. (2022). MiR-29a-3p improves acute lung injury by reducing alveolar epithelial cell PANoptosis. Aging Dis. 13, 899–909. 10.14336/AD.2021.1023 35656115 PMC9116916

[B9] Das B. Sarkar C. Rawat V. S. Kalita D. Deka S. Agnihotri A. (2021). Promise of the NLRP3 inflammasome inhibitors in *in vivo* disease models. Mol. (Basel, Switz.) 26, 4996. 10.3390/molecules26164996 PMC8399941 34443594

[B10] Feng S. Song G. Liu L. Liu W. Liang G. Song Z. (2022). Allergen-specific immunotherapy induces monocyte-derived dendritic cells but attenuates their maturation and cytokine production in the lesional skin of an atopic dermatitis mouse model. J. dermatology 49, 1310–1319. 10.1111/1346-8138.16582 36117445

[B11] Grailer J. J. Canning B. A. Kalbitz M. Haggadone M. D. Dhond R. M. Andjelkovic A. V. (2014). Critical role for the NLRP3 inflammasome during acute lung injury. J. Immunol. 192, 5974–5983. 10.4049/jimmunol.1400368 24795455 PMC4061751

[B12] Gu W. Zeng Q. Wang X. Jasem H. Ma L. (2024). Acute lung injury and the NLRP3 inflammasome. J. Inflamm. Res. 17, 3801–3813. 10.2147/jir.s464838 38887753 PMC11182363

[B13] Gurung P. Li B. Subbarao Malireddi R. K. Lamkanfi M. Geiger T. L. Kanneganti T. D. (2015). Chronic TLR stimulation controls NLRP3 inflammasome activation through IL-10 mediated regulation of NLRP3 expression and caspase-8 activation. Sci. Rep. 5, 14488. 10.1038/srep14488 26412089 PMC4585974

[B14] Heo S. K. Yun H. J. Yi H. S. Noh E. K. Park S. D. (2009). Evodiamine and rutaecarpine inhibit migration by LIGHT via suppression of NADPH oxidase activation. J. Cell. Biochem. 107, 123–133. 10.1002/jcb.22109 19241441

[B15] Kan X. Chen Y. Huang B. Fu S. Guo W. Ran X. (2021). Effect of Palrnatine on lipopolysaccharide-induced acute lung injury by inhibiting activation of the Akt/NF-κB pathway. J. Zhejiang Univ. Sci. B 22, 929–940. 10.1631/jzus.b2000583 34783223 PMC8593526

[B16] Kelley N. Jeltema D. Duan Y. He Y. (2019). The NLRP3 inflammasome: an overview of mechanisms of activation and regulation. Int. J. Mol. Sci. 20, 3328. 10.3390/ijms20133328 31284572 PMC6651423

[B17] Ko H. C. Wang Y. H. Liou K. T. Chen C. M. Chen C. H. Wang W. Y. (2007). Anti-inflammatory effects and mechanisms of the ethanol extract of Evodia rutaecarpa and its bioactive components on neutrophils and microglial cells. Eur. J. Pharmacol. 555, 211–217. 10.1016/j.ejphar.2006.10.002 17109845

[B18] Latronico N. Eikermann M. Ely E. W. Needham D. M. (2024). Improving management of ARDS: uniting acute management and long-term recovery. Crit. care (London, Engl.) 28, 58. 10.1186/s13054-024-04810-9 PMC10893724 38395902

[B19] Lawrence T. (2009). The nuclear factor NF-kappaB pathway in inflammation. Cold Spring Harb. Perspect. Biol. 1, a001651. 10.1101/cshperspect.a001651 20457564 PMC2882124

[B20] Lee C. Liao J. Chen S. Yen C. Lee Y. Huang S. (2019a). Fluorine-modified rutaecarpine exerts cyclooxygenase-2 inhibition and anti-inflammatory effects in lungs. Front. Pharmacol. 10, 91. 10.3389/fphar.2019.00091 30792658 PMC6374341

[B21] Lee S. A. Lee S. H. Kim J. Y. Lee W. S. (2019b). Effects of glycyrrhizin on lipopolysaccharide-induced acute lung injury in a mouse model. J. Thorac. Dis. 11, 1287–1302. 10.21037/jtd.2019.04.14 31179071 PMC6531684

[B22] Leszczyńska K. Jakubczyk D. Górska S. (2022). The NLRP3 inflammasome as a new target in respiratory disorders treatment. Front. Immunol. 13, 1006654. 10.3389/fimmu.2022.1006654 36203607 PMC9531678

[B23] Li C. Li X. Shi Z. Wu P. Fu J. Tang J. (2022b). Exosomes from LPS-preconditioned bone marrow MSCs accelerated peripheral nerve regeneration via M2 macrophage polarization: involvement of TSG-6/NF-κB/NLRP3 signaling pathway. Exp. Neurol. 356, 114139. 10.1016/j.expneurol.2022.114139 35690131

[B24] Li G. X. Jiang X. H. Zang J. N. Zhu B. Z. Jia C. C. Niu K. W. (2022a). B-cell receptor associated protein 31 deficiency decreases the expression of adhesion molecule CD11b/CD18 and PSGL-1 in neutrophils to ameliorate acute lung injury. Int. J. Biochem. and Cell Biol. 152, 106299. 10.1016/j.biocel.2022.106299 36210579 PMC9484107

[B25] Li J. M. Li X. Chan L. W. C. Hu R. Zheng T. Li H. (2023). Lipotoxicity-polarised macrophage-derived exosomes regulate mitochondrial fitness through Miro1-mediated mitophagy inhibition and contribute to type 2 diabetes development in mice. Diabetologia 66, 2368–2386. 10.1007/s00125-023-05992-7 37615690

[B26] Li X. Mai K. Ai Q. (2024). Palmitic acid activates NLRP3 inflammasome through NF-κB and AMPK-mitophagy-ROS pathways to induce IL-1β production in large yellow croaker (Larimichthys crocea). Biochimica biophysica acta. Mol. Cell Biol. lipids 1869, 159428. 10.1016/j.bbalip.2023.159428 38029958

[B27] Li Z. Yang M. Peng Y. Gao M. Yang B. (2019). Rutaecarpine ameliorated sepsis-induced peritoneal resident macrophages apoptosis and inflammation responses. Life Sci. 228, 11–20. 10.1016/j.lfs.2019.01.038 30690081

[B28] Liao Z. Q. Jiang Y. N. Su Z. L. Bi H. L. Li J. T. Li C. L. (2021). Rutaecarpine inhibits doxorubicin-induced oxidative stress and apoptosis by activating AKT signaling pathway. Front. Cardiovasc. Med. 8, 809689. 10.3389/fcvm.2021.809689 35071368 PMC8766983

[B29] Liaqat A. Mason M. Foster B. J. Kulkarni S. Barlas A. Farooq A. M. (2022). Evidence-based mechanical ventilatory strategies in ARDS. J. Clin. Med. 11, 319. 10.3390/jcm11020319 35054013 PMC8780427

[B30] Liu L. Zhang Y. Tang L. Zhong H. Danzeng D. Liang C. (2021). The neuroprotective effect of byu d mar 25 in LPS-induced alzheimer's disease mice model. Evidence-based complementary Altern. Med. eCAM 2021, 8879014. 10.1155/2021/8879014 PMC7936888 33727946

[B31] Liu M. Zhang Y. Yan J. Wang Y. (2022). Aerobic exercise alleviates ventilator-induced lung injury by inhibiting NLRP3 inflammasome activation. BMC Anesthesiol. 22, 369. 10.1186/s12871-022-01874-4 36456896 PMC9714243

[B32] Liu S. Su X. Pan P. Zhang L. Hu Y. Tan H. (2016). Neutrophil extracellular traps are indirectly triggered by lipopolysaccharide and contribute to acute lung injury. Sci. Rep. 6, 37252. 10.1038/srep37252 27849031 PMC5110961

[B33] Liu Z. J. Wang M. J. Luo J. Tan Y. T. Hou M. Wang S. C. (2023). A bibliometric analysis of hotpots and trends for the relationship between skin inflammation and regeneration. Front. Surg. 10, 1180624. 10.3389/fsurg.2023.1180624 37151861 PMC10160476

[B34] Luo J. Liu M. Wu X. Dou Y. Xia Y. Dai Y. (2015). DGAEE, a newly synthesized derivative of glycyrrhetinic acid, potently attenuates mouse septic shock via its main metabolite DGA in an IL-10-dependent manner. Int. Immunopharmacol. 29, 583–590. 10.1016/j.intimp.2015.09.025 26456500

[B35] Luo J. Wang L. Cui C. Chen H. Zeng W. Li X. (2024). MicroRNA-19a-3p inhibits endothelial dysfunction in atherosclerosis by targeting JCAD. BMC Cardiovasc. Disord. 24, 394. 10.1186/s12872-024-04063-y 39080547 PMC11287888

[B36] Luo J. Wang X. Jiang X. Liu C. Li Y. Han X. (2020). Rutaecarpine derivative R3 attenuates atherosclerosis via inhibiting NLRP3 inflammasome-related inflammation and modulating cholesterol transport. FASEB J. Fed. Am. Soc. Exp. Biol. 34, 1398–1411. 10.1096/fj.201900903RRR 31914630

[B37] Mazgaeen L. Gurung P. (2020). Recent advances in lipopolysaccharide recognition systems. Int. J. Mol. Sci. 21, 379. 10.3390/ijms21020379 31936182 PMC7013859

[B38] Mezzasoma L. Schmidt-Weber C. B. Fallarino F. (2023). *In vitro* study of TLR4-NLRP3-inflammasome activation in innate immune response. Methods Mol. Biol. (Clift. N.J.) 2700, 163–176. 10.1007/978-1-0716-3366-3_9 37603180

[B39] Niesler U. Palmer A. Radermacher P. Huber-Lang M. S. (2014). Role of alveolar macrophages in the inflammatory response after trauma. Shock (Augusta, Ga.) 42, 3–10. 10.1097/SHK.0000000000000167 24667621

[B40] Ozaki E. Campbell M. Doyle S. L. (2015). Targeting the NLRP3 inflammasome in chronic inflammatory diseases: current perspectives. J. Inflamm. Res. 8, 15–27. 10.2147/JIR.S51250 25653548 PMC4303395

[B41] Pelegrin P. Barroso-Gutierrez C. Surprenant A. (2008). P2X7 receptor differentially couples to distinct release pathways for IL-1beta in mouse macrophage. J. Immunol. 180, 7147–7157. 10.4049/jimmunol.180.11.7147 18490713

[B42] Proell M. Gerlic M. Mace P. D. Reed J. C. Riedl S. J. (2013). The CARD plays a critical role in ASC foci formation and inflammasome signalling. Biochem. J. 449, 613–621. 10.1042/bj20121198 23110696 PMC3966062

[B43] Qiao Y. Wang P. Qi J. Zhang L. Gao C. (2012). TLR-induced NF-κB activation regulates NLRP3 expression in murine macrophages. FEBS Lett. 586, 1022–1026. 10.1016/j.febslet.2012.02.045 22569257

[B44] Qin L. Q. Gu Z. Y. Chen N. Y. Liu P. D. Lan L. S. Li X. W. (2024). Rutaecarpine analogues with potent anti-inflammation to alleviate acute ulcerative colitis *via* regulating TLR4/MAPK/NF-κB pathway. Results Chem. 7, 101330. 10.1016/j.rechem.2024.101330

[B45] Qiu H. Chen X. Luo Z. Zhao L. Zhang T. Yang N. (2018). Inhibition of endogenous hydrogen sulfide production exacerbates the inflammatory response during urine-derived sepsis-induced kidney injury. Exp. Ther. Med. 16, 2851–2858. 10.3892/etm.2018.6520 30214506 PMC6125834

[B46] Song Y. X. Li X. Nie S. D. Hu Z. X. Zhou D. Sun D. Y. (2023). Extracellular vesicles released by glioma cells are decorated by Annexin A2 allowing for cellular uptake via heparan sulfate. Cancer gene Ther. 30, 1156–1166. 10.1038/s41417-023-00627-w 37231059

[B47] Su M. Hu R. Tang T. Tang W. Huang C. (2022). Review of the correlation between Chinese medicine and intestinal microbiota on the efficacy of diabetes mellitus. Front. Endocrinol. 13, 1085092. 10.3389/fendo.2022.1085092 PMC9905712 36760813

[B48] Su Y. N. Wang M. J. Yang J. P. Wu X. L. Xia M. Bao M. H. (2023). Effects of Yulin Tong Bu formula on modulating gut microbiota and fecal metabolite interactions in mice with polycystic ovary syndrome. Front. Endocrinol. 14, 1122709. 10.3389/fendo.2023.1122709 PMC9939769 36814581

[B49] Sun P. Sun N. Yin W. Sun Y. Fan K. Guo J. (2019). Matrine inhibits IL-1β secretion in primary porcine alveolar macrophages through the MyD88/NF-κB pathway and NLRP3 inflammasome. Veterinary Res. 50, 53. 10.1186/s13567-019-0671-x PMC6626430 31300043

[B50] Tian K. M. Li J. J. Xu S. W. (2019). Rutaecarpine: a promising cardiovascular protective alkaloid from Evodia rutaecarpa (Wu Zhu Yu). Pharmacol. Res. 141, 541–550. 10.1016/j.phrs.2018.12.019 30616017

[B51] Tsikis S. T. Fligor S. C. Hirsch T. I. Pan A. Yu L. J. Kishikawa H. (2022). Lipopolysaccharide-induced murine lung injury results in long-term pulmonary changes and downregulation of angiogenic pathways. Sci. Rep. 12, 10245. 10.1038/s41598-022-14618-8 35715592 PMC9205148

[B52] Wang L. Zhao M. (2022). Suppression of NOD-like receptor protein 3 inflammasome activation and macrophage M1 polarization by hederagenin contributes to attenuation of sepsis-induced acute lung injury in rats. Bioengineered 13, 7262–7276. 10.1080/21655979.2022.2047406 35266443 PMC9208453

[B53] Wang Z. Xu G. Li Z. Xiao X. Tang J. Bai Z. (2022). NLRP3 inflammasome pharmacological inhibitors in Glycyrrhiza for NLRP3-driven diseases treatment: extinguishing the fire of inflammation. J. Inflamm. Res. 15, 409–422. 10.2147/JIR.S344071 35082510 PMC8784972

[B54] Wei S. Ma W. Li X. Jiang C. Sun T. Li Y. (2020). Involvement of ROS/NLRP3 inflammasome signaling pathway in doxorubicin-induced cardiotoxicity. Cardiovasc. Toxicol. 20, 507–519. 10.1007/s12012-020-09576-4 32607760

[B55] Wu Y. X. He H. Q. Nie Y. J. Ding Y. H. Sun L. Qian F. (2018). Protostemonine effectively attenuates lipopolysaccharide-induced acute lung injury in mice. Acta Pharmacol. Sin. 39, 85–96. 10.1038/aps.2017.131 29047459 PMC5758663

[B56] Xu A. Deng F. Chen Y. Kong Y. Pan L. Liao Q. (2020). NF-κB pathway activation during endothelial-to-mesenchymal transition in a rat model of doxorubicin-induced cardiotoxicity. Biomed. and Pharmacother. = Biomedecine and Pharmacother. 130, 110525. 10.1016/j.biopha.2020.110525 32702633

[B57] Xu Y. Liu Q. Xu Y. Liu C. Wang X. He X. (2014). Rutaecarpine suppresses atherosclerosis in ApoE-/- mice through upregulating ABCA1 and SR-BI within RCT. J. lipid Res. 55, 1634–1647. 10.1194/jlr.m044198 24908654 PMC4109758

[B58] Yao J. Sterling K. Wang Z. Zhang Y. Song W. (2024). The role of inflammasomes in human diseases and their potential as therapeutic targets. Signal Transduct. Target. Ther. 9, 10. 10.1038/s41392-023-01687-y 38177104 PMC10766654

[B59] Ye Z. Wang P. Feng G. Wang Q. Liu C. Lu J. (2022). Cryptotanshinone attenuates LPS-induced acute lung injury by regulating metabolic reprogramming of macrophage. Front. Med. 9, 1075465. 10.3389/fmed.2022.1075465 PMC9880059 36714100

[B60] Yi J. Li L. Yin Z. J. Quan Y. Y. Tan R. R. Chen S. L. (2023). Polypeptide from moschus suppresses lipopolysaccharide-induced inflammation by inhibiting NF-κ B-ROS/NLRP3 pathway. Chin. J. Integr. Med. 29, 895–904. 10.1007/s11655-023-3598-z 37542626

[B61] Yu Z. Y. Cheng G. (2023). Protective effect of liriodendrin against liver ischaemia/reperfusion injury in mice via modulating oxidative stress, inflammation and nuclear factor kappa B/toll-like receptor 4 pathway. Folia Morphol. 82, 668–676. 10.5603/FM.a2022.0049 35607873

[B62] Yuan H. Perry C. N. Huang C. Iwai-Kanai E. Carreira R. S. Glembotski C. C. (2009). LPS-induced autophagy is mediated by oxidative signaling in cardiomyocytes and is associated with cytoprotection. Am. J. physiology. Heart circulatory physiology 296, H470–H479. 10.1152/ajpheart.01051.2008 PMC2643899 19098111

[B63] Zhang H. Zhu K. Zhang X. Ding Y. Zhu B. Meng W. (2022). Rutaecarpine ameliorates lipopolysaccharide-induced BEAS-2B cell injury through inhibition of endoplasmic reticulum stress via activation of the AMPK/SIRT1 signaling pathway. Exp. Ther. Med. 23, 373. 10.3892/etm.2022.11300 35495603 PMC9019775

[B64] Zhang J. Liu X. Wan C. Liu Y. Wang Y. Meng C. (2020). NLRP3 inflammasome mediates M1 macrophage polarization and IL-1β production in inflammatory root resorption. J. Clin. periodontology 47, 451–460. 10.1111/jcpe.13258 31976565

[B65] Zheng D. Liwinski T. Elinav E. (2020). Inflammasome activation and regulation: toward a better understanding of complex mechanisms. Cell Discov. 6, 36. 10.1038/s41421-020-0167-x 32550001 PMC7280307

[B66] Zito G. Buscetta M. Cimino M. Dino P. Bucchieri F. Cipollina C. (2020). Cellular models and assays to study NLRP3 inflammasome biology. Int. J. Mol. Sci. 21, 4294. 10.3390/ijms21124294 32560261 PMC7352206

