# Supplementary material for: 5-deoxy-rutaecarpine protects against LPS-induced acute lung injury via inhibiting NLRP3 inflammasome-related inflammation
Source: Front Pharmacol. 2025 Jan 28;16:1522146. doi: 10.3389/fphar.2025.1522146 (PMC11841402; doi:10.3389/fphar.2025.1522146)
Supplement: Supplementary file 1 [file Table1.docx]

**Table S1** Source of primary and secondary antibodies used for Western blotting.

| Antibodies | Supplier | Dilution |
| --- | --- | --- |
| IL-1β | Cell Signaling Technology | 1:1, 000 (in 5%BSA) |
| Caspase-1 | HuaBio | 1:1, 000 (in 5%BSA) |
| NLRP3 | Cell Signaling Technology | 1:1, 000 (in 5%BSA) |
| ASC | Cell Signaling Technology | 1:1, 000 (in 5%BSA) |
| p-IκBα | Bioworld | 1:1, 000 (in 5%BSA) |
| IκBα | Bioworld | 1:1, 000 (in 5%BSA) |
| p-p65 | Bioworld | 1:1, 000 (in 5%BSA) |
| p65 | Bioworld | 1:1, 000 (in 5%BSA) |
| Lamin B1 | HuaBio | 1:5, 000 (in 5%BSA) |
| GAPDH | HuaBio | 1:5, 000 (in 5%BSA) |
| IKKβ | Cell Signaling Technology | 1:1, 000 (in 5%BSA) |
| Goat Anti-Rabbit IgG H&L (HRP) | Zenbio | 1:8, 000 (in 1×TBST) |
| Goat Anti-Mouse IgG H&L (HRP) | Zenbio | 1: 8, 000 (in 1×TBST) |
