# Supplementary figures and images for: 5-deoxy-rutaecarpine protects against LPS-induced acute lung injury via inhibiting NLRP3 inflammasome-related inflammation
Source: Front Pharmacol. 2025 Jan 28;16:1522146. doi: 10.3389/fphar.2025.1522146 (PMC11841402; doi:10.3389/fphar.2025.1522146)

Uncropped western blots

Fig. 4A

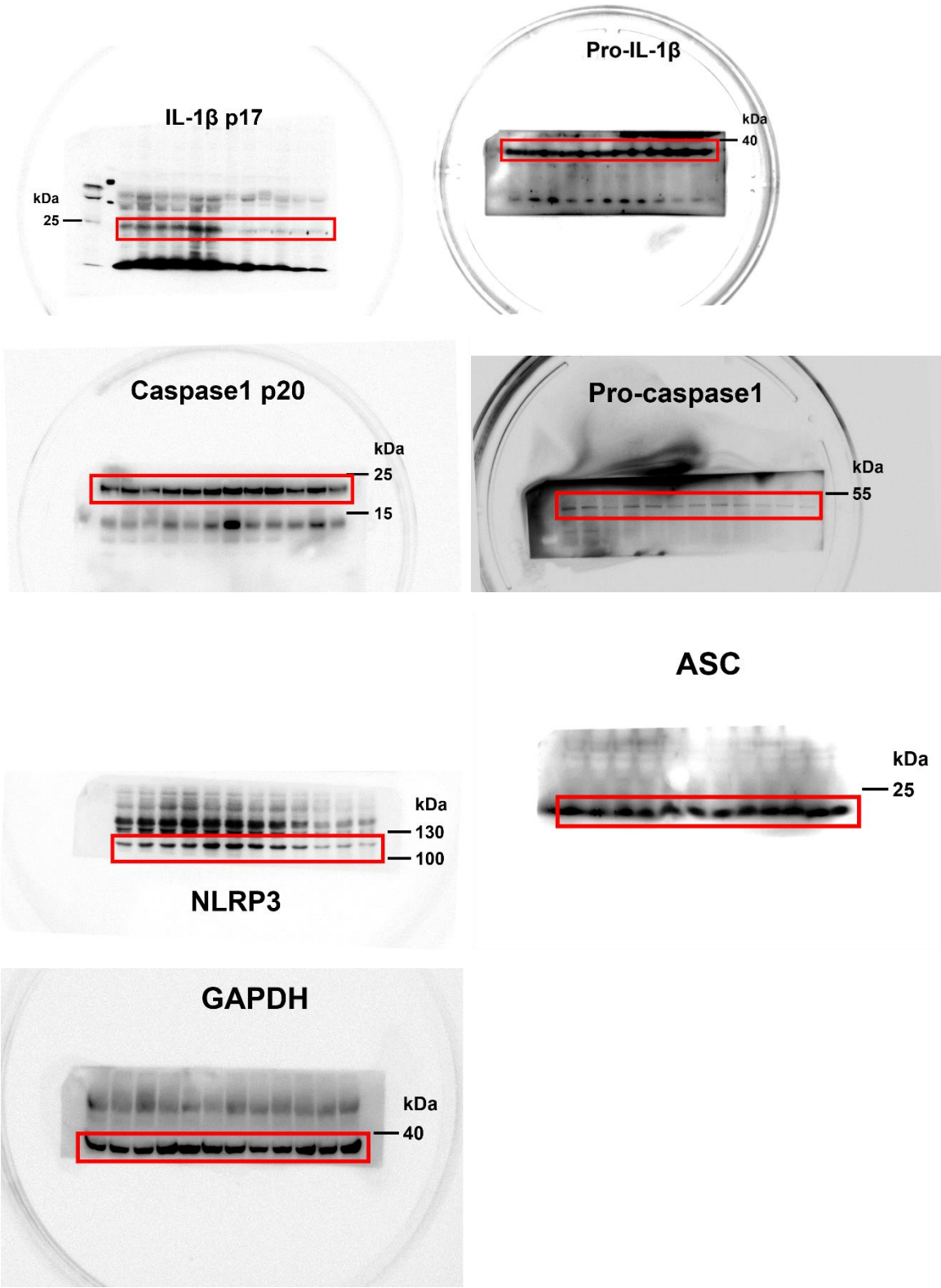

Fig. 5A

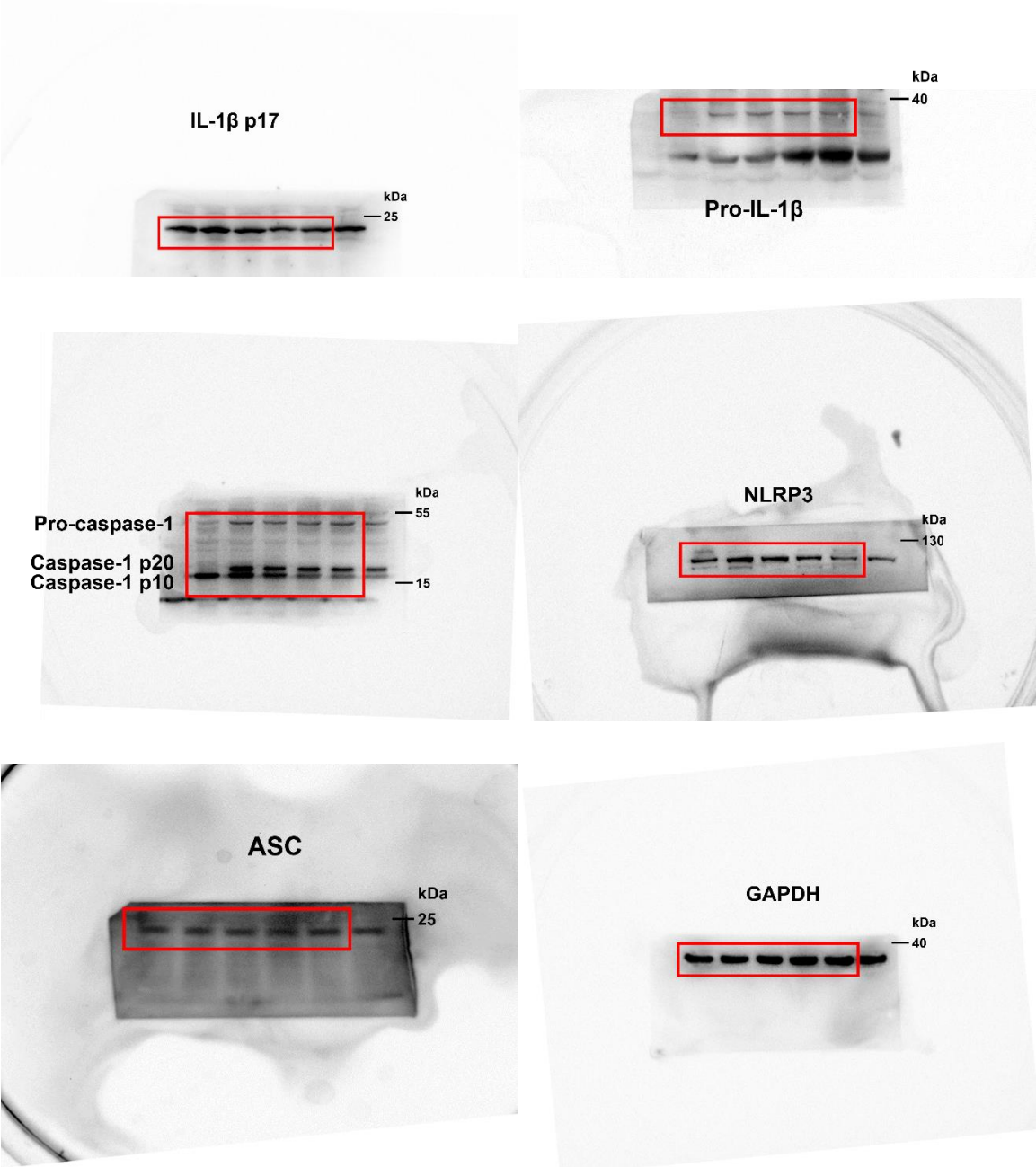

Fig. 6A

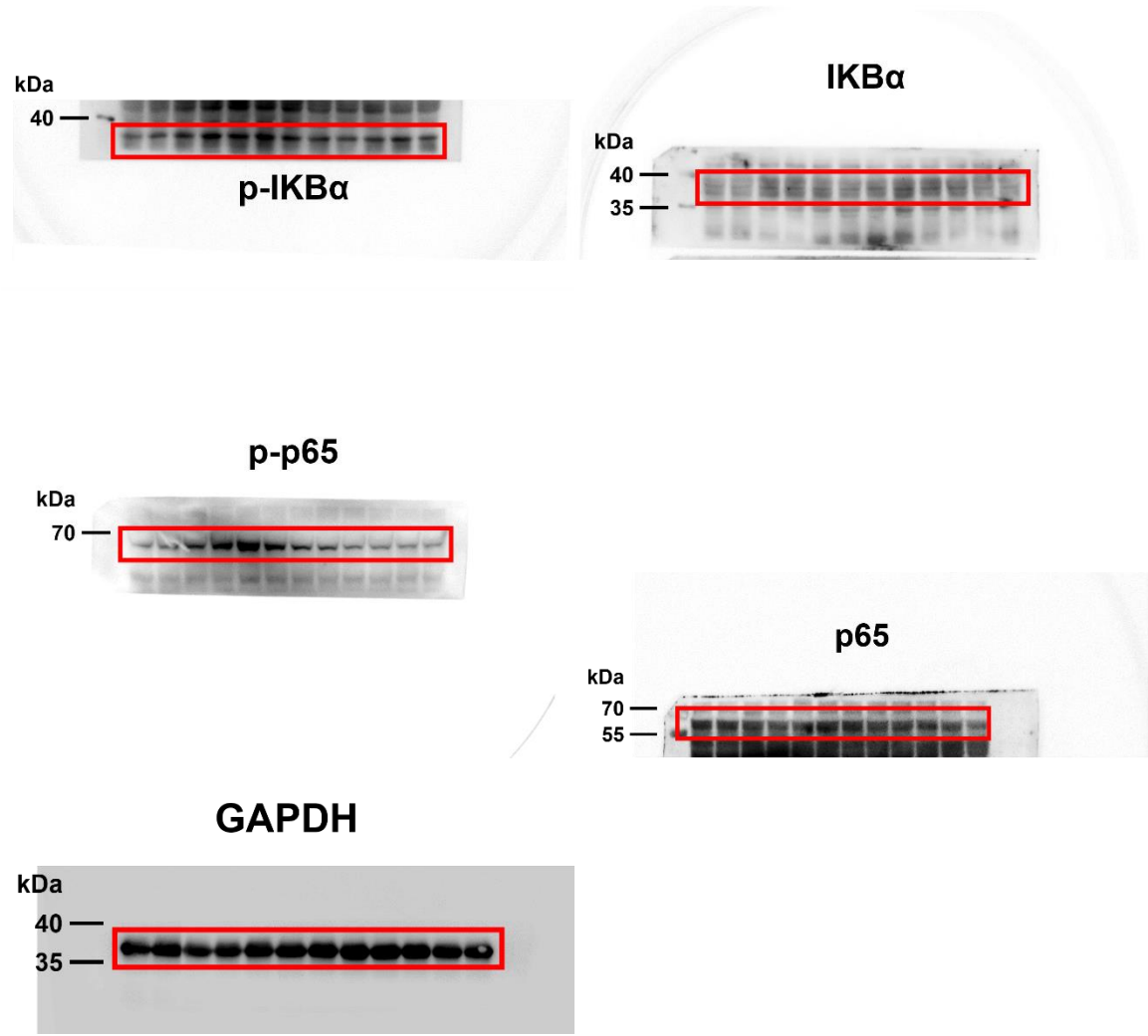

Fig. 7A

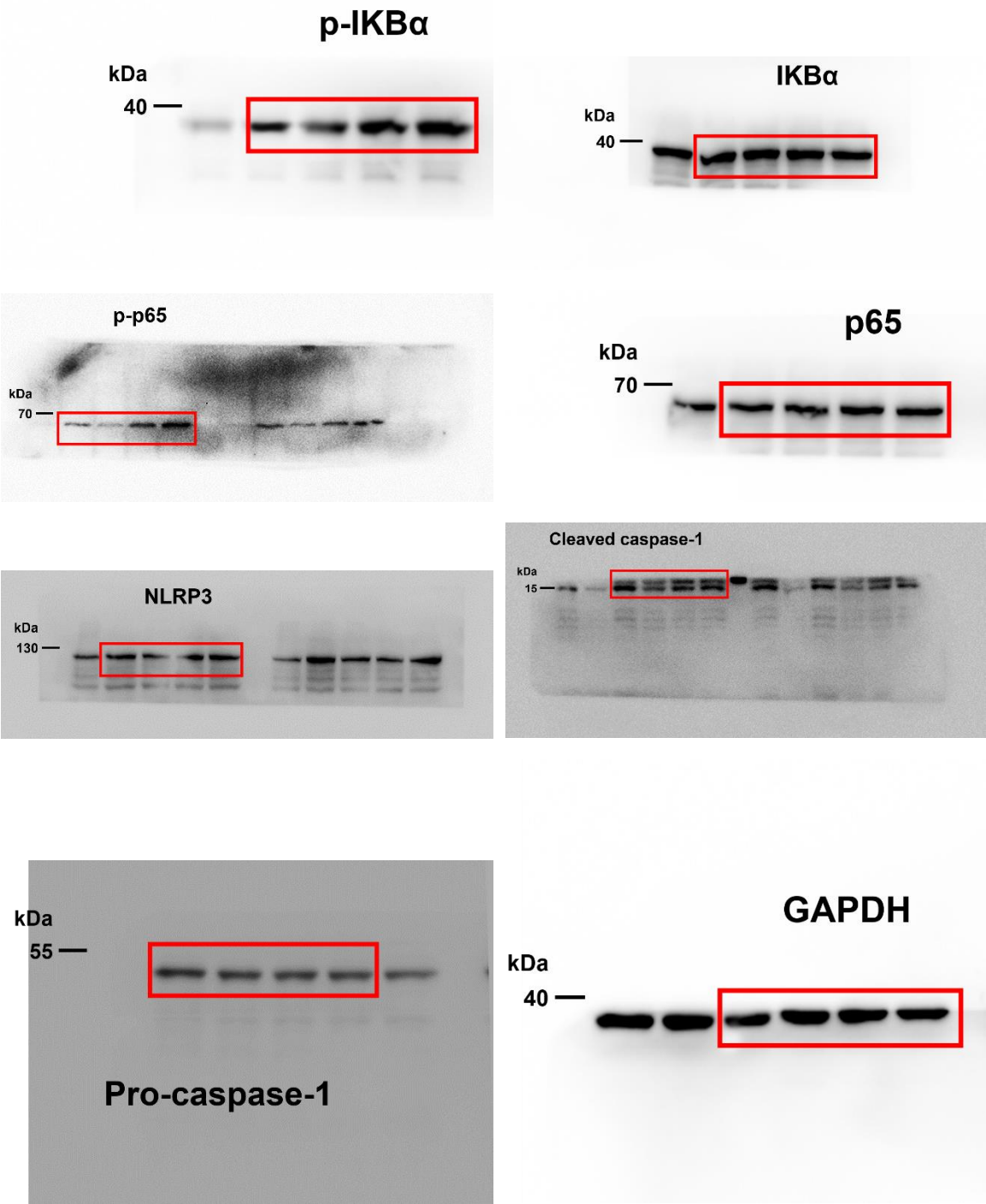

Fig. S1

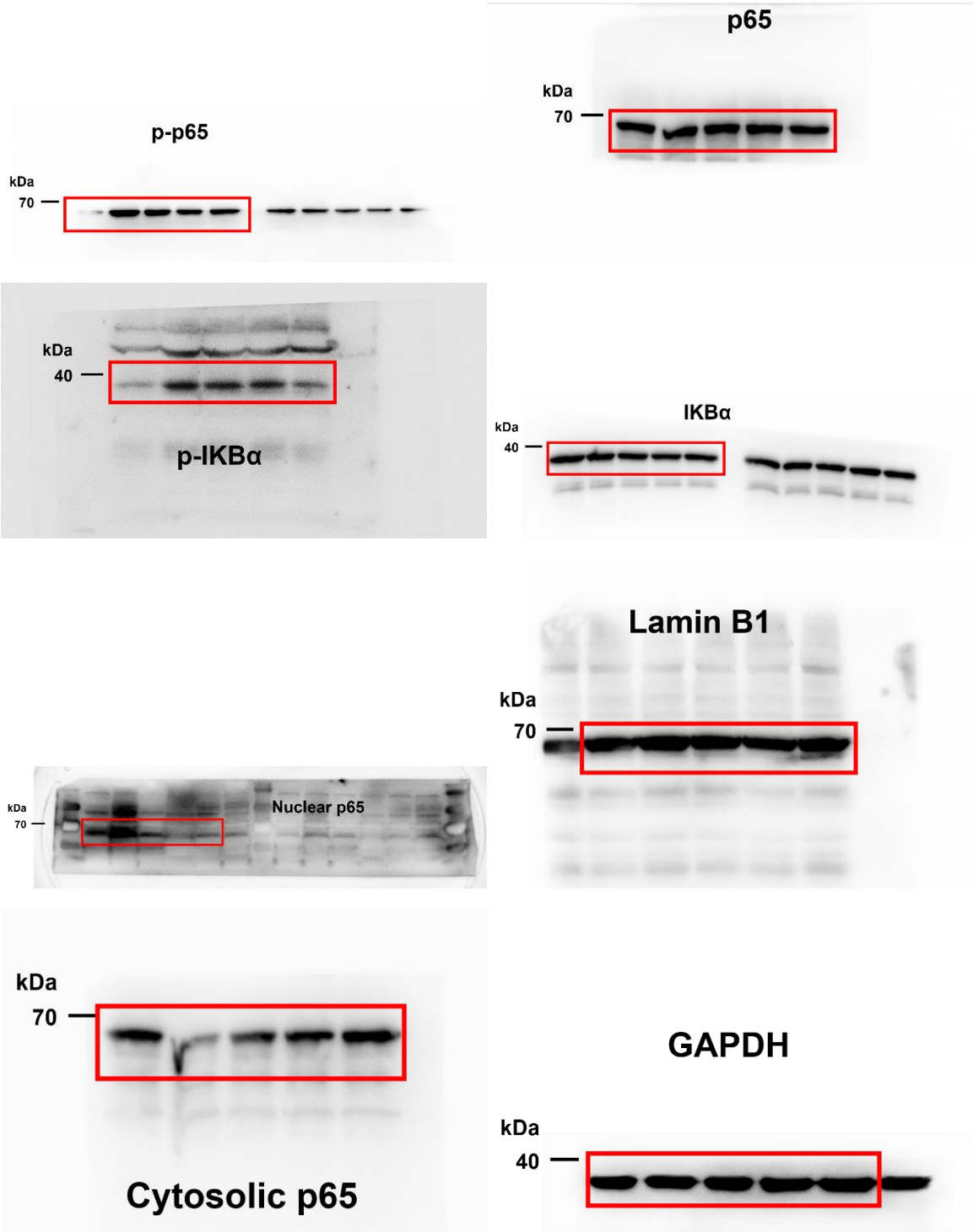

Fig. S2

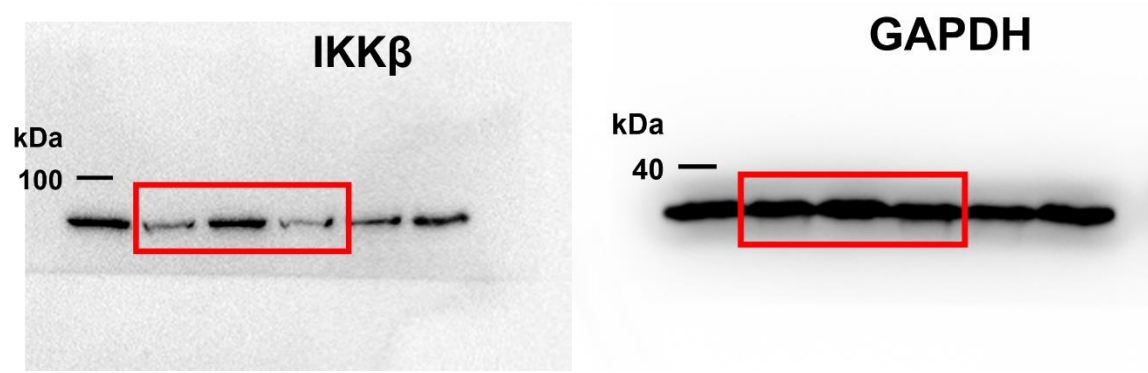

Supplement: Supplementary file 2 [file DataSheet1.pdf]
