# Supplementary material for: 5-deoxy-rutaecarpine protects against LPS-induced acute lung injury via inhibiting NLRP3 inflammasome-related inflammation
Source: Front Pharmacol. 2025 Jan 28;16:1522146. doi: 10.3389/fphar.2025.1522146 (PMC11841402; doi:10.3389/fphar.2025.1522146)
Supplement: Supplementary file 3 [file Image1.pdf]

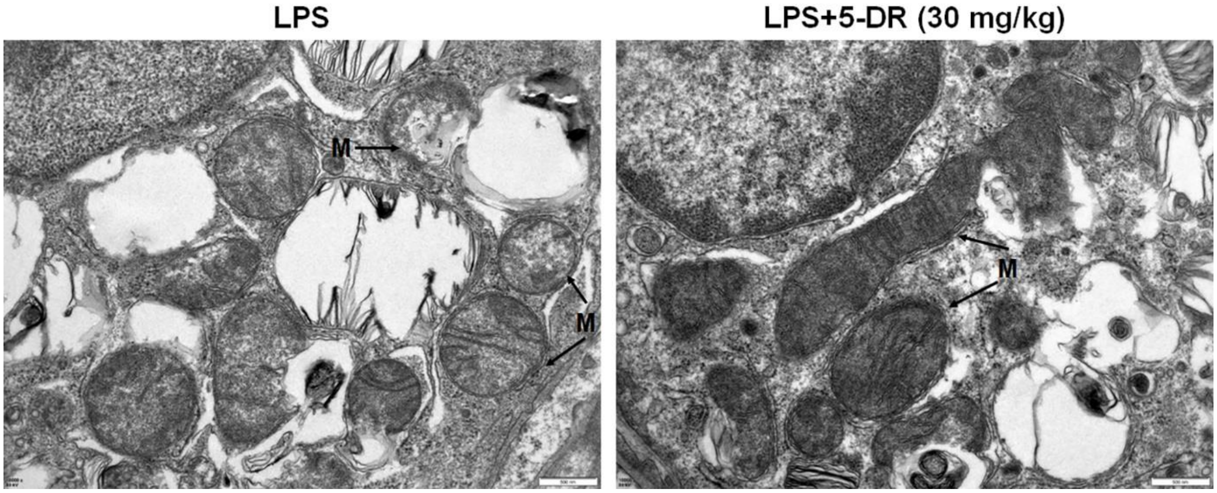


**Fig. S1** 5-DR attenuates the lung ultrastructural changes in LPS-challenged mice. C57BL/6 mice were administered with 5-DR (30 mg/kg) via gastric gavage once per day for 8 days before being treated by intratracheal instillation of LPS (5 mg/kg) for 24 h and then the lung tissues were isolated and cut into 1–2 mm3 cubes. Ultrastructural changes of lung tissues were observed under transmission electron microscope (10000×). Scale bar: 500 nm. M: mitochondria.

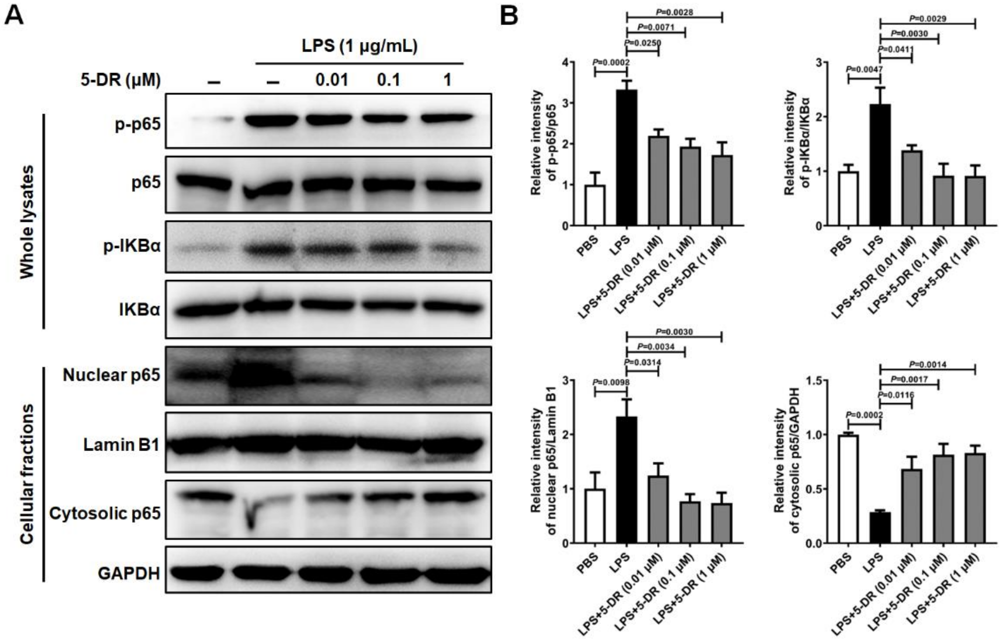


**Fig. S2** 5-DR inhibited NF-κB signaling and p65 transcription in J774A.1 cells. J774A.1 cells were pre-incubated with different concentrations of 5-DR (0.01, 0.1, 1 μM) for 20 h, then stimulated with LPS (1 μg/mL) for 4 h. Western blotting analysis was performed to evaluate the protein expression levels of whole cell proteins (p-p65, p65, p-IκBα, and IκBα), nuclear p65, and cytoplasmic protein p65. Results were presented as mean ± SEM (n = 3). Statistical analysis was performed by one-way ANOVA plus Dunnett's multiple comparisons test.

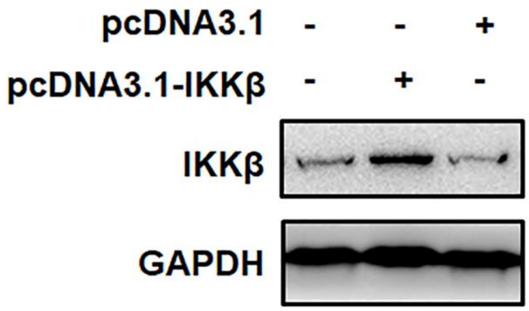


**Fig. S3** Western blotting analysis following transfection of J774A.1 cells with pcDNA3.1-IKKβ overexpressing plasmids (4 μg/well) or pcDNA3.1 plasmids (4 μg/well) prior to 5-DR or LPS treatment.
